# Marine facies differentiation along complex paleotopography: an example from the Middle Miocene (Serravallian) of Lower Austria

**DOI:** 10.1186/s00015-022-00425-w

**Published:** 2022-12-12

**Authors:** Werner E. Piller, Gerald Auer, Hugo Graber, Martin Gross

**Affiliations:** 1 Institute of Earth Sciences, University of Graz, NAWI Graz Geocenter, Heinrichstraße 26, 8010 Graz, Austria; 2 Carduccistraße 14, 39042 Brixen, BZ Italy; 3 Department for Geology & Palaeontology, Universalmuseum Joanneum, Weinzöttlstraße 16, 8045 Graz, Austria

**Keywords:** Paleoecology, Carbonates, Foraminifera, Ostracodes, Calcareous nannoplankton

## Abstract

In the area of Bad Deutsch-Altenburg (Hainburg Mountains, Lower Austria) a Middle Miocene transgression over Mesozoic basement was explored in the course of the Danube power plant project “Hainburg”. The Mesozoic basement forms a narrow ridge dipping to the northeast towards the Vienna Basin, covered by various Miocene sediments. The ridge represents a specific paleotopography that required a detailed study with 78 shallow, fully cored drill holes in an area of c. 0.5 km^2^. Ten drillings were selected for this study based on sedimentary composition and position relative to the Mesozoic ridge. These 10 cores, ranging in drilling depth from 26.5 to 96.4 m, were studied in respect to sedimentology, corallinacean algae, calcareous nannoplankton, foraminifers and ostracodes to reconstruct sediment distribution and paleoenvironment. Sediment distribution clearly shows that the Mesozoic ridge formed a physical barrier with siliciclastics dominating in the SW of the ridge and carbonate sediments prevailing in the NE. Based on biostratigraphy (calcareous nannoplankton, foraminifera, ostracodes, dinoflagellates) the majority of the sediments can be dated to the late Badenian (early Serravallian) only in some drillholes lower Sarmatian (upper Serravallian) sediments were detected. In terms of sequence stratigraphy, the Badenian sediments represent the transgressive and highstand systems tract of 3^rd^ order sequence TB 2.5 (bound by the lowstands Ser 2 and Ser 3), the lower Sarmatian sediments can be correlated to sequence TB 2.6. Carbonate sediments show a wide spectrum of 13 facies which are mostly dominated by coralline algae. According to the relative positions of the drill holes a water depth between 0 and about 50 m can be reconstructed what is supported by the occurrence of the benthic biota. This biota indicates that the sedimentary succession started from the very beginning under full marine conditions. Except of basal conglomerates/breccias water energy conditions were low and turbidity high. Close to the Sarmatian boundary a reduction in salinity and depth may have occurred which is also observed in the Sarmatian sediments. Carbonate sediments and, in particular, larger benthic foraminifers indicate tropical to warm-temperate conditions for the late Badenian of the studied sections. The siliciclastic sediments NW of the Mesozoic ridge reflect riverine input indicated by the occurrence of freshwater ostracodes and characean oogonias. Calcareous nannoplankton and dinoflagellates show a high share of reworking from Upper Cretaceaous and Paleogene sediments.

**Supplementary Information:**

The online version contains supplementary material available at 10.1186/s00015-022-00425-w.

## Introduction

The Paratethys Sea originated when the Western Tethys was split by the rising Alpine chains into the Proto-Mediterranean Sea in the south and the Paratethys Sea in the north (e.g., Kováč et al., 2007; Rögl, 1998, 1999; Steininger & Wessely, 2000). This separation occurred around the Eocene/Oligocene boundary and the connections of both realms changed frequently in time with temporarily active gateways between the Paratethys and the Proto-Mediterranean, the North Sea and the Indian Ocean but suffered from paleogeographic isolation during intervals in between (Rögl, 1999). The Paratethys itself is subdivided into a Western/Central Paratethys reaching from the Gulf of Lyon in the west to the Carpathian Mountains in the east and the Eastern Paratethys covering the area east of the Carpathian Mountains in the west and extended in the east to modern Lake Aral. The Central Paratethys was predominantly marine during the Oligocene to Middle Miocene and changed to the long-lived Lake Pannon during the early Late Miocene (Harzhauser & Piller, 2007; Steininger & Wessely, 2000).

The varying connections of the Paratethys with other oceans and seas resulted in a specific paleogeographic and paleoenvironmental development which necessitated the establishment of a regional chronostratigraphic scheme which is based mostly on endemic biota which cannot always easily be correlated to the global chronostratigraphy. Correlation out of the Paratethys is based on planktic foraminifera and calcareous nannoplankton and, to a minor extent, on magnetostratigraphy and sequence stratigraphy. Most of the biota, e.g., foraminifera, dinoflagellates, ostracodes, molluscs, and echinoids, are well studied in the Central Paratethys (e.g., Cicha et al., 1998; Harzhauser & Piller, 2007; Kollmann, 1960; Kroh, 2007; Soliman et al., 2022; Studencka et al., 1998).

**Fig. 1:**
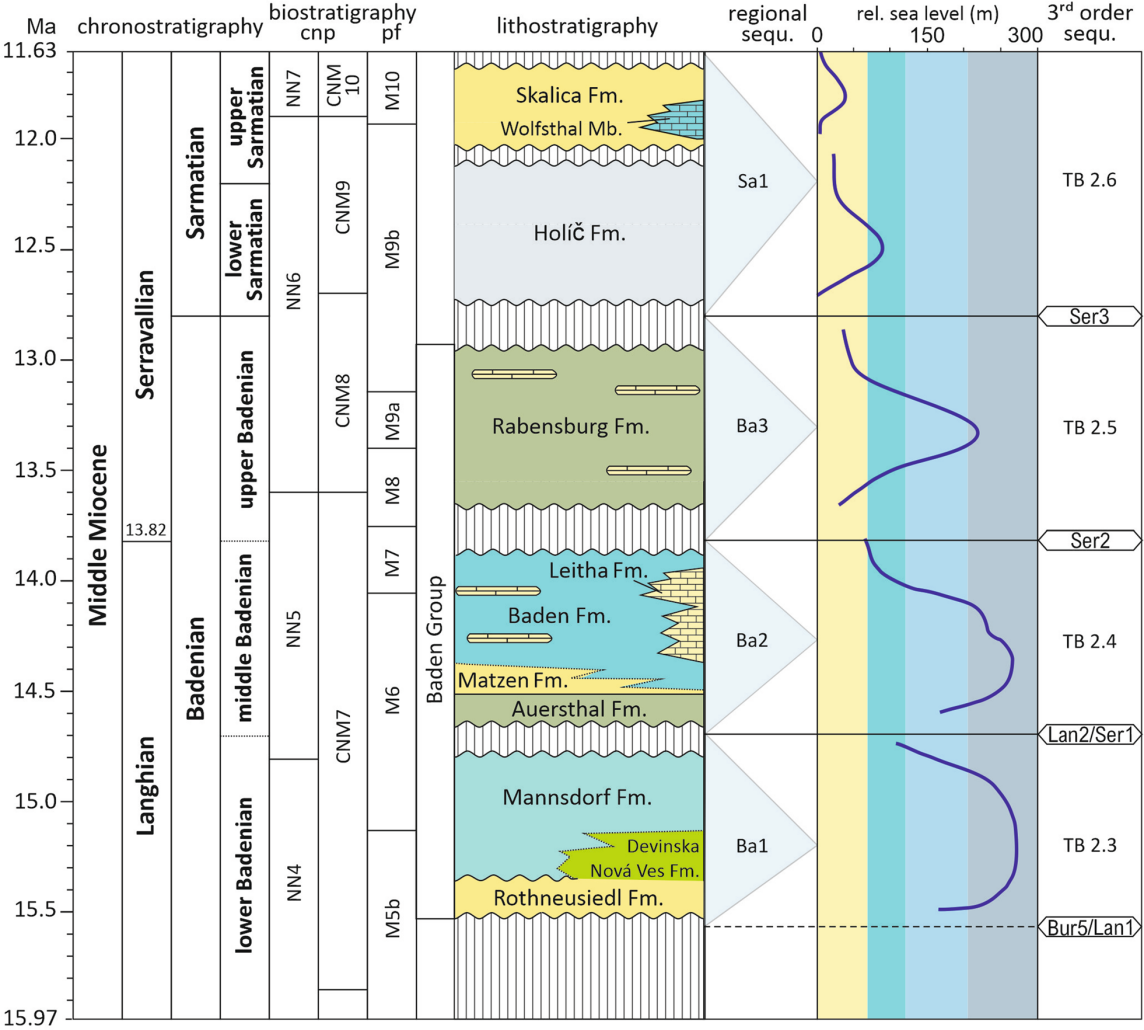
Stratigraphic chart of the Middle Miocene following the International Stratigraphic Chart (https://stratigraphy.org/chart) with the regional stages and substages of the Central Paratethys; the biostratigraphic columns show the calcareous nannoplankton (cnp) zonation after Martini (1971) (left) and after Backman et al. (2012) (right) and the planktic foraminiferal (pf) zonation after Wade et al. (2011); the lithostratigraphy follows Harzhauser et al. (2020), regional sequences after Strauss et al. (2006) and Siedl et al. (2020); the relative sea level indications in the Central Paratethys follow Kranner et al. (2021a) and the 3^rd^ order sequences Hardenbol et al. (1998)

The Badenian stage (Middle Miocene, Langhian-lower Serravallian; Fig. 1) is the biodiversity hotspot in the Central Paratethys (Harzhauser & Piller, 2007) documenting good water exchange with the Mediterranean but also favorable conditions related to the Middle Miocene Climatic Optimum (MMCO). Within the Badenian, however, also a climatic decline into the Middle Miocene Climate Transition (MMCT) occurs which is reflected in a reduction in biota diversity and composition. This very general development is frequently obscured by a strong facies differentiation with highly variable and localized fossil biota. In this study we will elaborate in a short time interval of the late Badenian and early Sarmatian an area which is facially highly diverse at a scale of few 100s of meters with very specific biota in space and time. Generally, the late Badenian is not very well studied in the Vienna Basin compared to the middle Badenian but in our example from Bad Deutsch-Altenburg, Lower Austria, we studied carbonate sedimentology, coralline red algae, foraminifera, ostracodes and calcareous nannoplankton to provide an integrated approach and a comprehensive paleoenvironmental reconstruction as well as a review on paleogeography.

## Geological background of the Vienna Basin

The Vienna Basin is one of the best studied pull apart basins in the world based on the occurrence of one of the largest hydrocarbon reservoirs in Europe (Arzmüller et al., 2006; Boote et al., 2018; Hamilton et al., 2000; Rupprecht et al., 2019). Originally, the basin was thought to have originated from simple pull apart kinematics (e.g., Royden, 1985, 1988) but its development is much more complex starting with a series of piggy-back basins at the front of the N- to NW-propagating thrust belt of the Eastern Alps. This initial phase is also called Proto-Vienna Basin which developed during the Early Miocene (Decker, 1996; Fodor, 1995; Lee & Wagreich, 2017). In the Middle to Late Miocene the basin was dominated by extensional tectonics due to lateral extrusion of the Eastern Alps (Decker, 1996; Fodor, 1995; Hölzel et al., 2010; Lee & Wagreich, 2017). This caused distinct fault systems, which subdivided the Vienna Basin into a complex horst and graben system producing several sub-basins (Hamilton et al., 2000; Hölzel et al., 2010; Kröll & Wessely, 1993; Siedl et al., 2020; Wessely, 2006).

The basin extends c. 200 km in N–S direction and c. 55 km west–east mostly on Austrian territory but also into the Czech Republic and the Slovak Republic. The latter parts are also well studied and documented in a number of publications (e.g., Baráth et al., 1994; Brzobohatý & Stráník, 2012; Buday, 1946; Fordinál et al., 2012; Kováč et al., 2004, 2007; Rybár et al., 2019; Sant et al., 2020; Špička, 1966; Vass, 2002; Vass et al., 1988). Sedimentation started in the Early Miocene (Ottnangian) and continued to the early Late Miocene (Pannonian). The maximum sediment thickness reaches about 5.500 m in the Schwechat deep. The complex tectonics with fault bounded uplifted blocks and depressions makes correlation within the Vienna Basin difficult. This tectonic pattern also produced a complex facies distribution within the basin additionally complicated by synsedimentary tectonics (e.g., Harzhauser et al., 2017, 2019; Siedl et al., 2020). The interplay of complex regional tectonics and global/regional sea level changes (Piller et al., 2007; Strauss et al., 2006) is also expressed in a complex lithostratigraphy which has been recently revised (Harzhauser et al., 2019, 2020). Many deep drillings in the basin and seismic lines allowed intra-basinal correlation by wire logs and biostratigraphy; the latter, however, represents a poorly constrained ecobiostratigraphy (cf. Harzhauser et al., 2020). Correlation with open oceanic equivalents is mostly based on planktic foraminifers and calcareous nannoplankton but also on cyclostratigraphy and astronomical tuning (Hohenegger & Wagreich, 2012; Hohenegger et al., 2009; Lirer et al., 2009).

During the Badenian (Langhian-early Serravallian) good water exchange with the Mediterranean Sea via the Slovenian “Trans-Tethyan Trench Corridor” (Bistricic & Jenk, 1985) was enabled what is also reflected by the highest biodiversity in the Miocene of the Central Paratethys (Harzhauser & Piller, 2007). This open connection is well expressed in the Vienna Basin by the recognition of three 3^rd^ order sequences allowing to subdivide the Badenian sediments into a lower, middle, and upper substage (Fig. 1). The base of the Badenian is characterized by a hiatus which is caused, in addition to the global sea level lowstand Bur5/Lan1, by the so called Styrian Tectonic Phase (Stille, 1924) and represents the lower Badenian Ba1 sequence (Strauss et al., 2006) which can be correlated to the 3^rd^ order sequence TB 2.3 of Hardenbol et al. (1998) ending again with a hiatus. The middle Badenian is reflected by the hiatus bounded sequence Ba2 (TB 2.4) and the upper Badenian by sequence Ba3 (TB 2.5) starting again with a hiatus representing the lowstand Ser2. The Badenian/Sarmatian boundary is again characterized by a major sea level drop and a subsequent restriction from the Mediterranean resulting in reduced biodiversity (Harzhauser & Piller, 2007; Fig. 1). Paleoenvironmental conditions changed strongly through the Badenian (and Sarmatian) guided by strong tectonics and paleoclimate (Kranner et al., 2021a, 2021b; Siedl et al., 2020). Besides the application of planktic foraminifers and calcareous nannoplankton for correlation to the Mediterranean Miocene, within the Vienna Basin an ecobiostratigraphic zonation was developed (Grill, 1941) and repeatedly modified but more recently proposed to be abandoned (Kranner et al., 2021a, 2021b). The mostly used zonation consists of the Lower Lagenid Zone, Upper Lagenid Zone, the *Spirorutilus* Zone and the *Bulimina-Bolivina* Zone. The Lagenid zones cover the lower Badenian and lower part of the middle Badenian, the *Spirorutilus* Zone the middle Badenian and the *Bulimina-Bolivina* Zone the upper Badenian. Both, definition and stratigraphic extent of these zones are poorly constrained.

## Study area

**Fig. 2:**
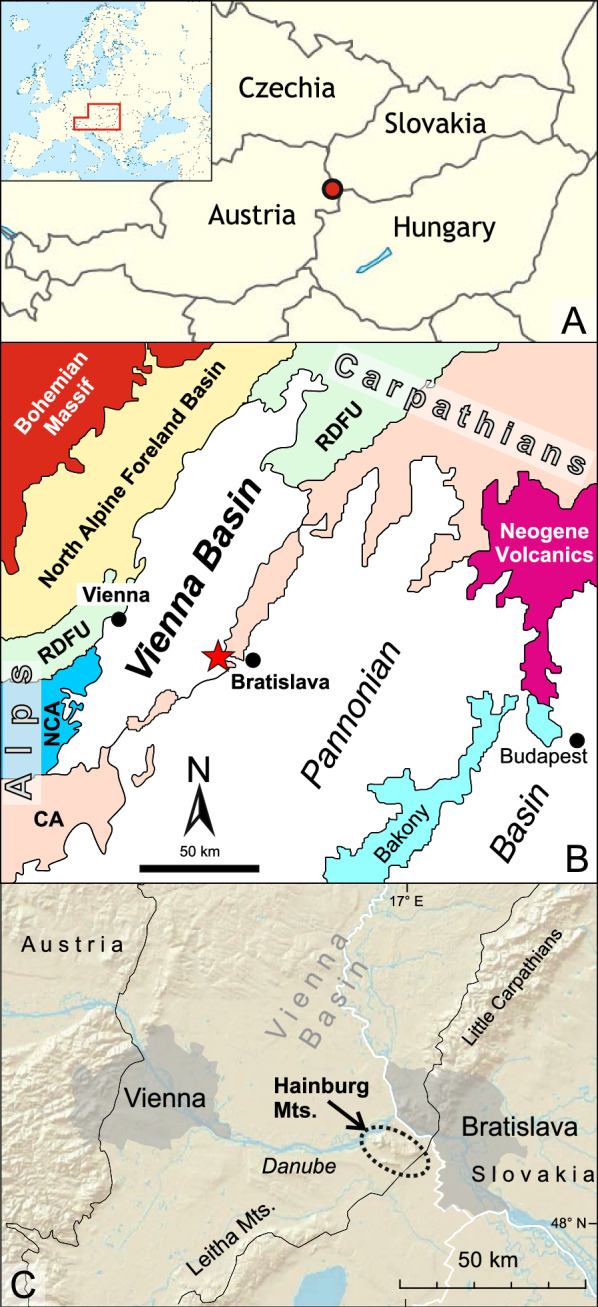
**A** General location maps showing the study area. **B** Simplified geological map with the position of the Vienna Basin related to the Alpine-Carpathian Mountains and the Pannonian Basin (modified after Wessely, 1998). **C** Topographic map (DEM) of the Central Vienna Basin (between Vienna and Bratislava) with the Hainburg Mountains indicated as well as the Little Carpathians and the Leitha Mountains; *RDFU* Rhenodanubian Flysch Unit, *NCA* Northern Calcareous Alps, *CA* Central Alps

The studied area is located in the Hainburg Mountains (Lower Austria) which mark the easternmost margin of the Vienna Basin but also the boundary to the Danube Basin in the east (Fig. 2). The latter is part of the Pannonian Basin System and exhibits a diverging origin from the Vienna Basin. The Hainburg Mountains represent the southern end of the Little Carpathians and belong geologically to the tatrid unit (Fig. 2). They are made up of granites, gneiss and mica schists, and Permo-Triassic quartzites overlain by Middle Triassic limestone and dolomite (Kristan-Tollmann & Spendlingwimmer, 1975; Wessely, 1961). These rocks are occasionally covered by Badenian and Sarmatian sediments. A detailed description of the geology of the Hainburg Mountains is provided by Wessely (1961) who also studied the Miocene sediments on top of the crystalline core and Mesozoic cover. He described a transgression of Badenian sediments onto the basement along a steep shoreface with coralline algal limestones and marls in more distal positions. Biostratigraphically he classified these sediments as part of the *Spirorutilus*- and *Bulimina-Bolivina* zones.

**Fig. 3:**
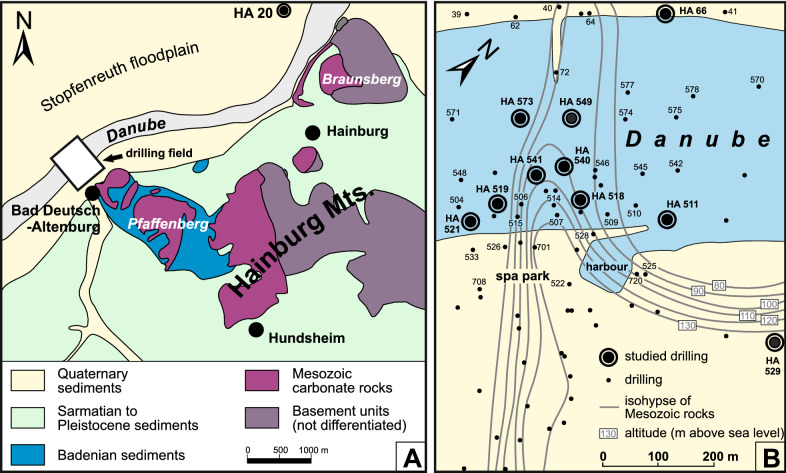
**A** Simplified geological map of the Hainburg Mountains between Bad Deutsch-Altenburg and Hainburg (modified after Fuchs, 1985) and the location of the drilling field. **B** The drilling field at Bad Deutsch-Altenburg with all drillings and the studied drill sites indicated; the contour lines (isohypses) reflect the morphology of the Triassic dolomite ridge (modified after Gangl, 1988)

In the reconnaissance phase for the Danube powerplant project “Hainburg” possible impacts on the mineral springs at Bad Deutsch-Altenburg have been studied in great detail. For this sensitive topic a dense grid of 78 shallow, fully cored boreholes have been drilled in the spa area and its surroundings as well as in the Danube riverbed and at the left riverbank mostly located in the municipality of Bad Deutsch-Altenburg (Gangl, 1988, 1990; Fig. 3) representing an area of only c. 0.5 km^2^. These drillings penetrated the Quaternary-Miocene sediments and some boreholes also reached the Mesozoic basement. The specific geological situation which necessitated this detailed study is a Mesozoic dolomite ridge which crops out at the Kirchenberg and Pfaffenberg in Bad Deutsch-Altenburg but dips to the northwest into the deeper part of the Vienna Basin (Fig. 3). This ridge is onlapped and overlain by Badenian sediments which show small scale lateral and vertical facies variations. Above a transgression conglomerate the Triassic dolomite is overlain by coralline algal limestones in the literature referred to as “Leitha Limestone” or “Leitha Limestone Facies” (Gangl, 1988, 1990; Wessely, 1961, 2006). Overall, the Badenian sediments in the southwest of the Triassic spur are dominated by sandy and marly sediments while in the northeast of the spur coralline algal limestones prevail. This special situation makes this location interesting for studying various aspects. So far, the focus was placed mainly on the study of ostracodes with a first study and new description of taxa by Danielopol et al. (1991). A comprehensive study on ostracodes was carried out by Gross (2002) but only partly published (Gross, 2006). In accordance with the data from Wessely (1961) most of the samples (59) were assigned to the upper Badenian only a few (7) from the top of two boreholes were placed into the lower Sarmatian (HA 66, HA 573). The Badenian samples indicate full marine conditions, in the samples SW of the Triassic ridge the ostracode assemblages indicate freshwater input, the assemblages NE of the spur indicate an epi- to mesoneritic environment (Gross, 2002).

## Methods

For studying isolated foraminifera and ostracodes small rock pieces were treated with diluted hydrogen peroxide (H_2_O_2_:H_2_O = 1:4; 30%) and washed over a sieve set of 2 mm, 1 mm, 500 µm, 250 µm, 125 µm, and 63 µm for size fractionation. Microfossils were picked under binocular microscopes. The fractions > 250 µm were completely picked or at least 200 individuals extracted. Smaller fractions have been screened not to miss important biotic constituents. For SEM studies (Zeiss LEO, DSM 982) selected specimens were treated with dish detergent and subsequently cleaned in an ultrasonic bath. Adhering sediment particles were removed with preparation needles.

Calcareous nannofossil slides were prepared according to an adapted preparation protocol derived from Bordiga et al. (2015) providing the possibility to perform absolute nannofossil abundance estimates. First, the samples were freeze-dried, and about 10 mg of sample were suspended in 50 ml of 1 M NH_3_ buffer solution. Afterwards, 1.5 ml of buffered suspension were transferred to a 24 × 42 mm coverslip, gently dried at 50 °C, and mounted using Norland Optical Adhesive No. 61^®^. All nannofossil slides were studied under 1000 × magnification using a Zeiss Axioplan2 microscope. Selected samples were also prepared for SEM analysis by transferring 750 µL onto a 18 × 18 mm coverslip.

For classifying carbonate rocks representative cores were cut in half, polished and photographed for studying the composition macroscopically. In addition, out of 30 samples 41 thin sections (5 × 5 cm) were prepared and studied under a binocular microscope (Zeiss SteREO Discovery.V20).

For measurements of the tests of the foraminiferal taxon *Amphistegina* from 3 boreholes (HA 66, HA 511, HA 540) 11 samples have been studied. Out of these 2552 individuals were picked and mounted on a plate with adhesive tape in positions parallel and vertical to the plate. In these positions diameter and thickness (besides other parameters) have been measured with a binocular microscope Leica MZ16 using the ImageAccess ver. 5 rel. 186. The resulting data are summarized in Table 1.

**Table 1:** Basic data of the 11 studied drillings; measurements in meters; m asl: meters above sea level; topics studied at the respective borehole: C: carbonate facies, F: foraminifers, O: ostracodes, N: calcareous nannoplankton

Borehole	HA 66	HA 511	HA 518	HA 519	HA 521	HA 529	HA 540	HA 541	HA 549	HA 573	HA 20
Altitude (m asl)	142.7	136.0	136.5	134.0	135.3	146.8	134.9	134.8	133.0	133.4	141.5
Quaternary thickness	13.1	3.8	3.3	3.7	4.8	8.1	3.4	3.8	4.0	4.2	12.4
Miocene thickness	78.7	31.2	33.1	26.3	21.7	23.7	29.2	25.2	32.4	69.8	37.4
Mesozoic thickness	4.6	-	4.1	-	-	9.2	5.0	3.0	2.9	5.1	-
Total thickness	96.4	35.0	40.5	30.0	26.5	41.0	37.6	32.0	39.3	79.1	49.8
Topics studied	C, F, O, N	C, F, O, N	C	O	O	O	C, F, O	O	O	F, O, N	O

## Results

### Studied cores

**Fig. 4:**
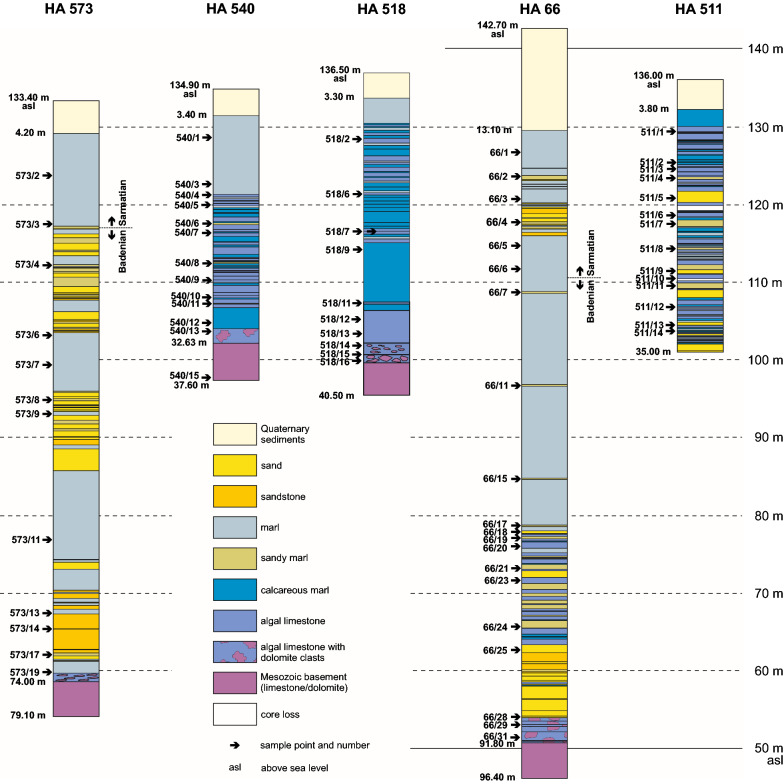
Lithologs of the detailed studied cores arranged according to their position in respect to altitude (asl) and roughly perpendicular to the axis of the Mesozoic ridge. Indicated for all drillings are sample locations (arrow), altitude above sea level (asl), depth of the Quaternary, depth of Mesozoic basement (if reached) and final depth

**Fig. 5:**
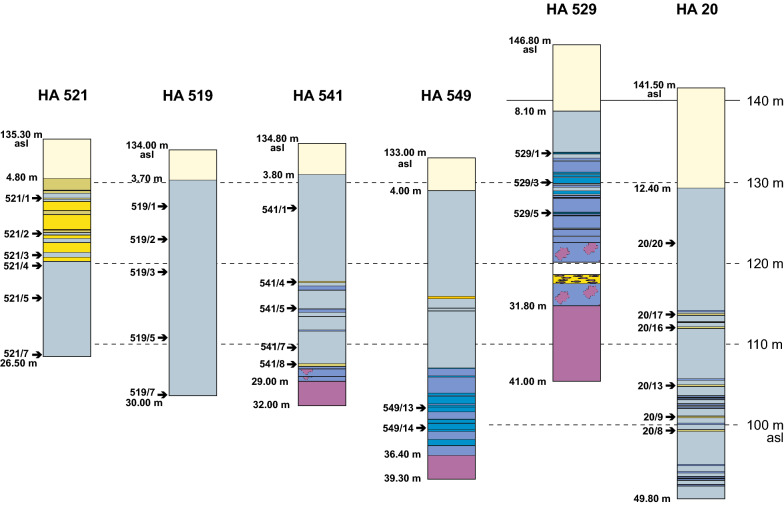
Lithologs of cores studied only for ostracodes. The logs are arranged according to their position in respect to altitude (asl) and roughly perpendicular to the axis of the Mesozoic ridge. Indicated for all drillings are sample locations (arrow), altitude asl, depth of the Quaternary, depth of Mesozoic basement (if reached) and the final depth

Altogether 11 cores with highly variable lithologies were studied. The cores, their lithologies and sample positions are shown in Figs. 4 and 5. These boreholes were selected being characteristic for a specific area and facies (i.e., from the crest of the Mesozoic ridge, siliciclastically dominated boreholes SW of the ridge, carbonate dominated cores from NE of the ridge). In 7 drill holes the Mesozoic basement was reached and 2 cores contain the Badenian/Sarmatian boundary (Ha 66, Ha 573). Basic data and studied topics are represented in Table 1. Location and spatial arrangement of the drillings are shown in Fig. 3.

### Carbonate facies

Four boreholes were studied in detail for carbonate facies which are located on the crest of the Mesozoic ridge (HA 518, HA 540) or penetrated the carbonate dominated sediments in the NE of the ridge (HA 66, HA 511) (Fig. 4).

**Fig. 6:**
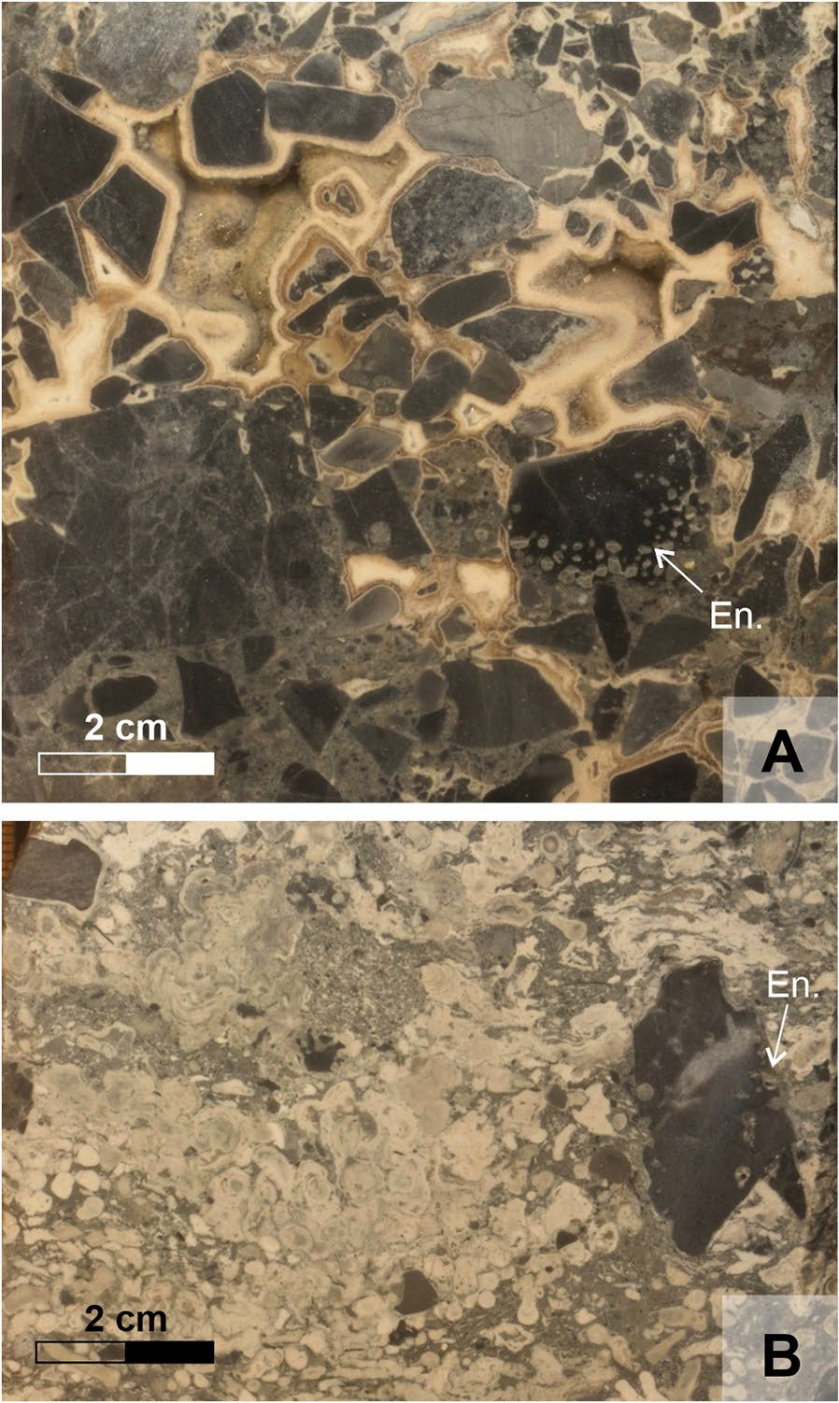
**A** Polished core surface of the transgression conglomerate of Triassic dolomite clasts lithified by several cement generations. Some of the components are bored by *Entobia* igen. (En.), the brownish/reddish coloration stems from pyrite crystals (sample HA 518/16). **B** Limestone sample immediately above the Triassic basement with bored (*Entobia* igen., En.) dolomite clasts, coralline algal encrusted clasts and rhodolites and branch fragments of coralline algae (polished core surface, sample HA 540/15)

Generally, the Triassic basement on which the Badenian transgression started is covered by a veneer of upper Badenian limestones occurring in the lower part of the structure on top of the crest but also along the flanks of the ridge, being in the higher parts restricted to the flanks (Fig. 14). The sediments which mark the immediate transgression horizon represent a coarse breccia or conglomerate composed of Triassic dolomite clasts which can reach nearly one meter in thickness. The basal sediments show predominantly a breccia without matrix and the components are lithified by thick cement crusts (HA 518/16; Fig. 6A). The dolomite clasts are bored by clionid sponges (*Entobia* igen.) and lithophagid bivalves (*Gastrochaenolites* igen.) clearly indicating a marine environment from the very base. These breccia components become further up incrusted by coralline algae and matrix starts to occur which gradually increases in volume (Fig. 6B). Components become smaller and the sediment gets matrix supported. The matrix is formed of small dolomite fragments, but increasingly coralline algal components, foraminifers and molluscs occur. In examples where a coarse breccia is missing corallinacean limestone overlies the dolomite at a sharp, wavy boundary and also as infills of karst cavities (HA 540/15). The margin of the karstic dolomite is bored by lithophagid bivalves and clinoid sponges.

The studied upper Badenian limestones show a broad spectrum in composition and facies. They range from pure limestones to marly limestones or sandy limestones with highly variable carbonate contents grading also into calcareous marl and even marl. The later have a high carbonate content which is due to a high amount of the larger benthic foraminifer *Amphistegina*. These occur in pure limestones but also in *Amphistegina* marls where they represent rock forming amounts sometimes in floatstone/packstone texture with marly/sandy matrix (see also below).

**Fig. 7:**
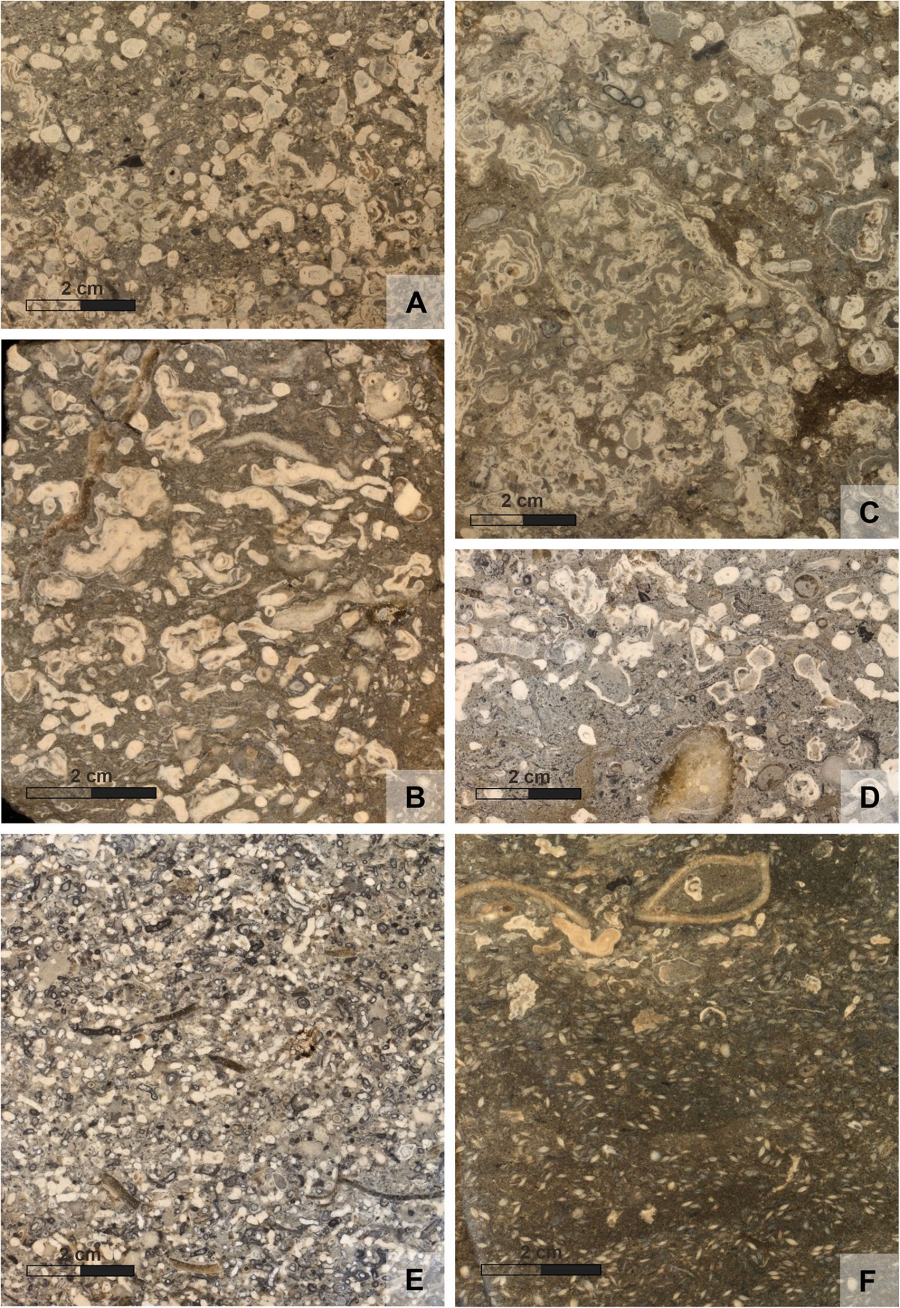
Characteristic facies of the carbonate rocks: **A** Branched coralline algal facies with few dolomitic lithoclasts (HA 518/12). **B** Branched and crustose coralline algal facies (HA 511/1). **C** Boxwork rhodolites facies (HA 518/9). **D** Bryozoan-coralline algal facies; branched bryozoan colonies are incrusted by coralline algae (HA 511/3). **E** Maërl facies with small but very abundant algal branch fragments (HA 511/1). **F** *Amphistegina* facies with densely packed, bioturbated *Amphistegina* mudstone and molluscs in the upper part (HA 66/20)

**Fig. 8:**
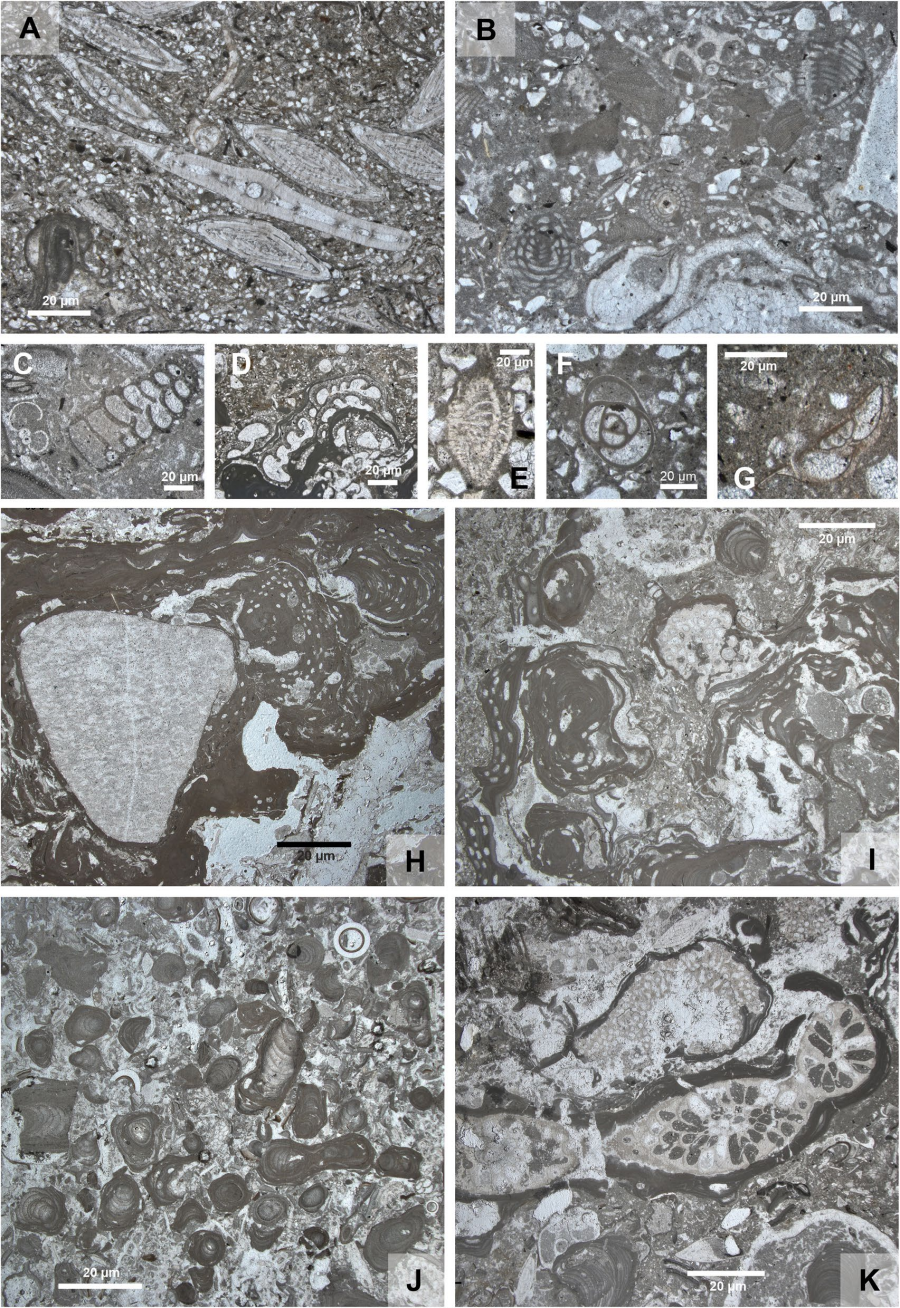
Foraminifera and coralline red algae in rock thin sections. **A** Fine grained *Amphistegina* terrigenous facies with tests of *Amphistegina* and *Planostegina* (center) (HA 66/20). **B** Bioclastic packstone with the larger foraminifer *Borelis melo* (HA 540/13). **C** Biserial agglutinated and planktonic foraminifera (HA 511/3). **D** Sesssile agglutinated foraminifer *Haddonia* (HA 540/). **E** Elphidiid foraminifer in subaxial section (HA 518/16). **F** Miliolid foraminifer (HA 540/13). **G** Cibicidid foraminifer in axial section (HA 511/6). **H** Multispecies rhodolite with a Triassic dolomite clast as nucleus (HA 518/2). **I** Multispecific boxwork rhodolite of warty/branched and crustose algal thalli with typical sediment-filled voids and borings (*Gastrochaenolites* igen.); intergrown are also bryozoan colonies (HA 540/7). **J** Branched corallinacean thalli representing maërl facies with agglutinated foraminifera, serpulids and scaphopods (511/1) **K** Bryozoan colonies encrusted by coralline algae with *Amphistegina*, molluscs and serpulids (HA 511/3)

The majority of the calcareous sediments are dominated by calcareous algae in various growth forms and preservation states. Based on these characters various facies types can be differentiated:

- Branched corallinacean facies: most dominating components are branches of coralline algal thalli up to a few centimeters length and bifurcation can also frequently be observed indicating that these are at least larger fragments (compared to the Maërl facies) (Fig. 7A).
- Maërl facies: this is a facies type made up of small fragments of branched corallinacean thalli which are mostly densely packed (rudstone) with subordinate other bioclasts, mainly molluscs (Fig. 7E). A gradual transition to the branched corallinacean facies occurs.
- Encrusting corallinacean facies: a corallinacean facies with dominant algal crusts which may encrust other components not or only subordinately forming rhodolites. They co-occur with branched algal thalli.
- Boxwork rhodolite facies: the characteristic and dominating components are boxwork rhodolites which are open rhodolites with a high amount of sediment between the algal laminae (Fig. 7C). The shape can vary from spherical to discoidal and the size gets up to 6 cm. In most examples a nucleus is not detectable except in the basal beds where dolomite clasts frequently form the nuclei.
- Terrigenous corallinacean facies: a variable amount of coralline algal fragments in a sandy to marly matrix (floatstones).
- Bryozoan-corallinacean facies: nodular and branching bryozoan colonies and fragments which are frequently encrusted by coralline algae (Figs. 7D, 8K). This facies is rare in the studied material.
- Mollusc-corallinacean facies: characteristic for this facies is the co-occurrence of molluscs and corallinacean algae.
- Corallinacean dolomite clasts facies: this facies is restricted to the basal parts of the boreholes where variable amounts of the underlying dolomite occur together with coralline algae. The latter may occur as branches or encrust the dolomite clasts (Fig. 8H).

Besides these corallinacean dominated facies a few others also occur:

- Terrigenous *Amphistegina* facies: *Amphistegina* dominated sediments (packstone, floatstone, rudstone) which frequently have a (sandy) marl matrix (Fig. 7F).
- Terrigenous *Amphistegina*-bivalve facies: in addition to the high amount of *Amphistegina* also molluscs occur in considerable amounts within a marly matrix.
- Terrigenous bryozoan facies: bryozoans occur in higher amounts in a (sandy) marl matrix.
- Dolomite breccia: this facies is restricted to the lowermost layers immediately above the Triassic basement (Fig. 6A).

All described facies may grade into each other not representing clearly defined types. In the vertical distribution of the facies no regular pattern is observable except that the dolomite influenced facies occur at or close to the Mesozoic basement.

### Coralline red algae

Coralline red algae (CRA) are major constituents of the carbonate rocks/sediments in the studied cores and this rock type is commonly called “Leitha limestone” or “Leitha Limestone Facies”. The Leitha limestone, formalized in the Leitha Formation by Harzhauser et al. (2020), is, however, of middle Badenian age. Although the first fossil coralline algae were described out of the Leitha Limestone of Neudörfl (today: Devínska Nová Ves; see also below) by Reuss (1847) the taxonomic inventory is still insufficiently documented. The widespread corallinacean algae in the Badenian of the Vienna Basin are generally poorly studied. Besides first mentions in the nineteenth century (e.g., Reuss, 1847; Unger, 1858) a more detailed description gave Conti (1946). A first detailed documentation of upper Badenian coralline red algae from the area around Devínska Nová Ves provided Schaleková (1969) and, more recently, Hrabovský (2013) who studied middle Badenian “Leitha limestones” of the area. In addition, Schaleková (1973) studied upper Badenian coralline algae further NNE (quarry at Rohožník-Vajar, Slovakia).

The studied material is with more than 10 taxa relatively diverse, including *Hydrolithon corculumis*, *Lithophyllum duplex*, *Lithophyllum lithothamnoides*, *Lithophyllum microsporum*, *Lithophyllum* spp., *Lithothamnion ramosissimum*, *L. minervae*, *Lithothamnion* spp., *Mesophyllum roveretoi*, *Mesophyllum* spp., *Neogoniolithon* sp., *Phymatolithon calcareum*, *Spongites fruticulosus*, *Spongites anguineum*, and *Sporolithon* spp. No regular vertical distribution pattern of the taxa within the cores has been recognized.

Growth forms of the thalli are highly variable (Woelkerling et al., 1993) and considered of ecological importance (e.g., Aguirre et al., 2017; Bosellini & Ginsburg, 1971). Here, growth forms terminology follows Woelkerling et al. (1993) represented by encrusting forms with warty to fruticose protuberances. These may occur encrusting a specific substratum, representing coatings of rock fragments or bioclasts (e.g., bryozoan). Also crustose layered to foliose thalli occur without overgrowth on hard substrate (Fig. 7B). Aggregates of non-attached coralline red algae, so called rhodolites (Aguirre et al., 2017; Riosmena-Rodríguez, 2017), are very abundant. Their size is highly variable ranging from < 1 to 6 cm (size observation is, however, limited by the core diameter and larger rhodoliths are to be expected). Their shapes are mostly subspherical to ellipsoidal and their surface is warty to fruticose (Fig. 8H), most abundant are boxwork rhodolites (Basso, 1998) (Figs. 7C, 8I). The end member of fragmentation and optional re-growth of thalli and rhodolites is a sediment called “maërl” which consists of densely packed coralline algal branches (Figs. 7E, 8J). Similar to taxonomic composition, growth forms exhibit no specific distributional pattern within the studied cores.

Some of the growth forms have been used for differentiating carbonate facies types (see above). The growth forms in these facies types may grade into each other but may consist predominantly of branched fragments or very densely packed branched fragments (“maërl”).

### Foraminifera

**Fig. 9:**
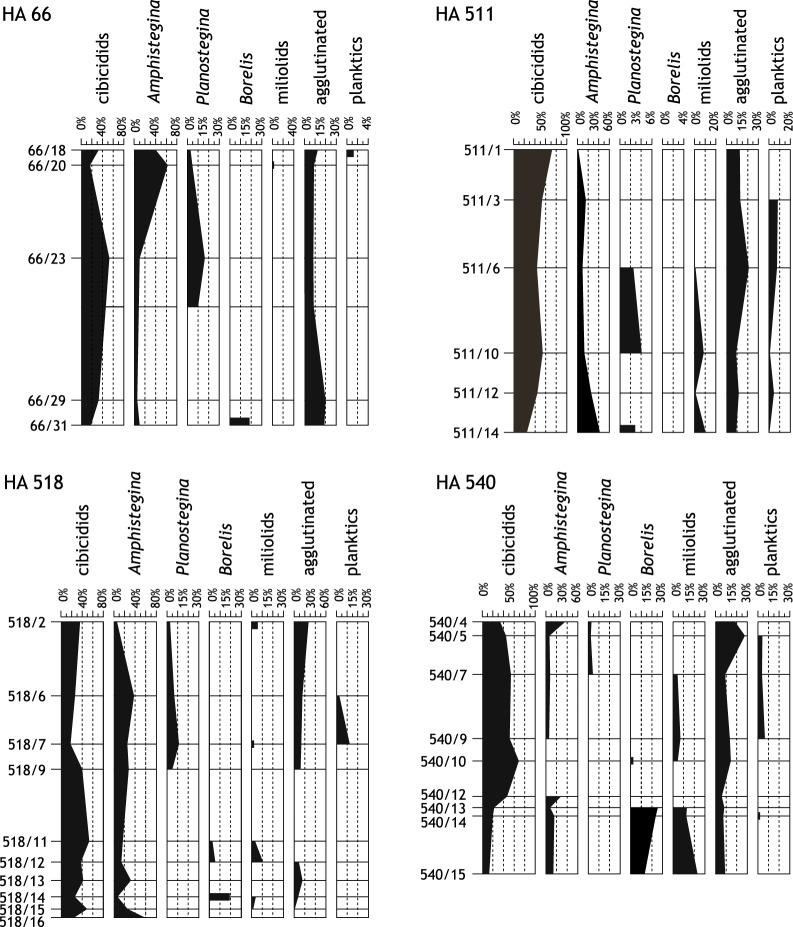
Distribution of selected foraminiferal taxa in thin sections of the 4 cores HA 66, HA 511, HA 518, and HA 540

Foraminifera were studied both in thin sections of limestones and on extracted isolated material from non-lithified sediment samples. Out of the limestones 41 thin sections of 30 samples were studied, the same as for carbonate facies (Figs. 7F, 8A–G, 9).

For foraminiferal analysis of the washed material 33 samples were studied out of 4 boreholes (HA 66, HA 511, HA 540, HA 573) and additional samples were screened for stratigraphic or paleoecologic markers. The preservation of the isolated foraminiferal tests is generally poor to moderate showing dissolution features, fragmention p.p., cemented sediment particles and the taphocoenoses are very incomplete compared to the original biocenoses. This can be concluded because agglutinated foraminifers are extremely rare in all washed samples and also miliolid taxa are rare but when preserved mostly in a very bad condition. Also, some hyaline taxa seem to be missing. This is in contrast to thin sections where both agglutinated and miliolid taxa occur in higher abundances (Figs. 8C, D, F, 9). Because of this diagenetic overprint of the assemblages neither a quantitative nor statistical approach could have applied to foraminifera. A semiquantitative recording was carried out in thin section analysis (Fig. 9; Tables 2, 3).

**Table 2:** *Amphistegina mammilla*: statistical parameters for the ratio diameter/thickness (d/t) of the tests

Sample number	Number of specimens	Mean d/t	Standard deviation	Minimum	Maximum
HA 66/19	260	2.68	0.43	1.53	4.20
HA 66/21	198	2.63	0.47	1.36	3.83
HA 66/24	195	2.74	0.52	1.23	4.45
HA 511/8	220	3.04	0.45	1.89	4.28
HA 511/9	201	2.88	0.37	1.97	4.58
HA 511/11	359	2.98	0.44	1.72	4.38
HA 511/13	286	2.79	0.46	1.40	4.32
HA 511/15	206	3.09	0.72	1.59	5.83
HA 540/6	205	2.89	0.43	1.93	4.31
HA 540/8	215	2.93	0.42	1.80	4.64
HA 540/11	207	2.69	0.39	1.65	3.80

**Table 3:** Foraminiferal abundance in thin sections of 30 samples, differentiating 7 taxa and 1 unidentified category (in percentage)

Sample nr	66/18	66/20	66/23	66/29	66/31	511/1	511/3	511/6	511/10	511/12	511/14	518/2	518/6	518/7	518/9
cibicidids	34.57	16.54	52.78	33.33	18.18	72.12	53.57	43.42	54.79	43.48	24.27	36.11	26.53	17.91	40.00
*Amphistegina*	38.27	63.78	8.33	6.67	9.09	0.96	14.29	9.21	12.33	26.09	40.78	5.56	38.78	26.87	28.0
*Planostegina*	2.47	4.72	16.67	0.00	0.00	0.00	0.00	2.63	4.11	0.00	2.91	2.78	7.14	11.94	4.00
*Borelis*	0.00	0.00	0.00	0.00	18.18	0.00	0.00	0.00	0.00	0.00	0.00	0.00	0.00	0.00	0.00
miliolids	0.00	0.79	0.00	0.00	0.00	0.00	0.00	1.32	8.22	1.45	10.68	5.56	0.00	1.49	0.00
agglutinated	12.35	8.66	8.33	20.00	18.18	12.50	12.50	21.05	8.22	11.59	8.74	27.78	15.31	13.43	12.00
planktics	1.23	0.00	0.00	0.00	0.00	0.00	7.14	7.89	1.37	4.35	0.97	0.00	2.04	13.13	0.00
unidentified	11.11	5.51	13.89	40.00	36.36	14.42	12.50	14.47	10.96	13.04	11.65	22.22	10.20	14.93	16.00
total number	81	127	36	15	11	104	56	76	73	69	103	36	98	67	25

Sample nr	518/11	518/12	518/13	518/14	518/15	518/16	540/4	540/5	540/7	540/9	540/10	540/12	540/13	540/14	540/15
cibicidids	53.57	37.84	42.11	26.92	49.32	25.00	32.39	45.45	54.35	51.72	69.77	47.06	22.67	20.00	12.28
*Amphistegina*	17.86	13.51	31.58	7.69	24.66	57.50	36.62	7.27	8.70	6.90	0.00	29.41	8.00	16.36	15.79
*Planostegina*	0.00	0.00	0.00	0.00	0.00	0.00	2.82	2.73	4.35	0.00	0.00	0.00	0.00	0.00	0.00
*Borelis*	3.57	5.41	0.00	19.23	0.00	0.00	0.00	0.00	0.00	0.00	2.33	0.00	25.33	23.64	15.79
miliolids	3.57	10.81	0.00	3.85	1.37	0.00	0.00	0.00	4.35	6.90	4.65	0.00	13.33	12.73	22.81
agglutinated	0.00	8.11	15.79	7.69	1.37	0.00	18.31	25.45	8.70	13.79	13.95	5.88	8.00	7.27	8.77
planktics	0.00	0.00	0.00	0.00	0.00	0.00	0.00	4.55	4.35	6.90	0.00	0.00	0.00	1.82	0.00
unidentified	21.43	24.32	10.53	34.62	23.29	17.50	9.86	14.55	15.22	13.79	9.30	17.65	22.67	18.18	24.56
total number	28	37	19	26	73	40	71	110	46	29	43	17	75	55	57

Overall, the most conspicuous taxon is the larger benthic foraminifer (LBF) *Amphistegina mammilla* (Figs. 8A, 9, 10.1a–c) which occurs in the studied cores in rock-forming quantities (Fig. 7F). This species occurs both in limestones (terrigenous *Amphistegina* facies, terrigenous *Amphistegina*-bivalve facies; Fig. 7F) and in fine siliciclastics mostly sandy *Amphistegina* marl or marly *Amphistegina* fine sand. They are rare in pure pelitic sediments. The limestones where they occur are also rich in siliciclastic sand and represent floatstones or packstones (Fig. 7F). In core HA 66 *Amphistegina* occurs only in the lower part of the borehole (HA 66/25 to HA 66/17) and is completely absent above even in the sandy marls. In HA 511 it occurs throughout the section but is rare in the sandy bryozoan marl (HA 511/7). Foraminifera are generally rare in HA 540 but HA 540/11, /8 and /6 are *Amphistegina* marls. In HA 573 *Amphistegina* is comparably rare, occurring only in sample HA 573/11. The measurements on the *Amphistegina* tests show maximum diameters (d) of about 3 mm and a thickness (t) of 1.37 mm. Sizes are distinctly smaller in samples HA 511/13 (max. d: 1.57 mm, max. t: 0.76 mm) and HA 511/15 (max. d: 1.66 mm, max. t: 0.6 mm). The average d/t ratio ranges between 2.63 and 3.09 (details are given in Table 1).

Another LBF is *Borelis melo* (Figs. 8B, 9, 10.2) which is well represented in the limestone facies occurring in HA 66/31, HA 518/14, /12, /11 and HA 540/15, /14, /13, /10. In washed samples only in HA 540/1 and HA 573/11 a few badly preserved specimens were detected.

The third LBF is *Planostegina* (Fig. 8A) which has not been found in washed samples but frequently in thin sections HA 66/23, /20, HA 511/14, /10, /6, /3, HA 518/9, /7, /6, HA 540/7, /5, /4. *Borelis* occurs predominantly immediately above the Mesozoic basement whereas *Planostegina* occurs only higher up in the section (Fig. 9).

Smaller foraminifera are nearly continuously represented by cibicidis (including *Lobatula lobatula* which cannot be consistently separated in thin section) (Figs. 8G, 9) and *Elphidium* spp. (Fig. 8E). *Lobatula* is relatively rare in HA 573 except of HA 573/19 from the base of the core, occurs throughout HA 511 and HA 540 and is rather rare in the upper part of HA 66. *Bolivina* spp. and *Bulimina* spp. (e.g., Fig. 10.3) are most abundant in the upper samples of HA 66, occur throughout HA 573, being abundant in HA 540/3 and nearly absent in HA 511. *Pappina neudorfensis* (Fig. 10.4) and *Uvigerina* spp. occur in the upper part of HA 66 (except HA 66/7) and in some samples of HA 573 but only in very low numbers. *Asterigerinata planorbis* is recorded in all cores in small quantities but is very rare in HA 511. All other benthic taxa are in subordinate quantities present/preserved.

**Fig. 10:**
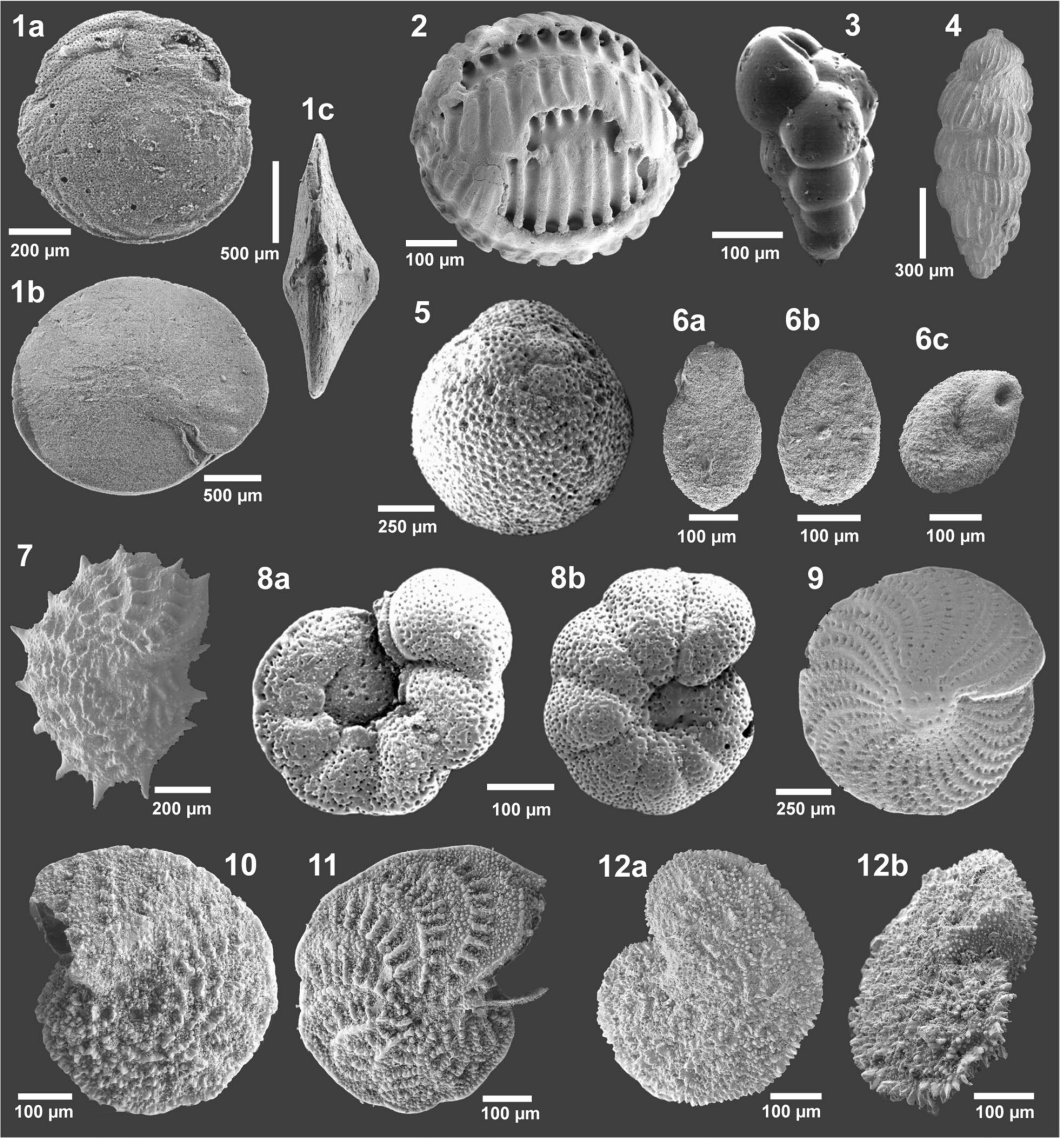
Isolated foraminifera from upper Badenian and lower Sarmatian sediments. 1. *Amphistegina mammilla* (Fichtel & Moll, 1798), a: spiral view, b: umbilical view, c: lateral view, upper Badenian, sample HA 511/8; 2. *Borelis melo* (Fichtel & Moll, 1798), upper Badenian, sample HA 540/1; 3. *Bulimina elongata* (d’Orbigny, 1826), lateral view, upper Badenian, sample HA 573/9; 4. *Pappina neudorfensis* (Toula, 1900), lateral view, upper Badenian, sample HA 66/25; 5. *Orbulina suturalis* Brönnimann, 1951, upper Badenian, sample HA 511/5; 6. *Saccammina sarmatica* Venglinsky, 1958, a, b: lateral views, c: apertural view, lower Sarmatian, sample HA 573/3; 7. *Elphidium aculeatum* (d’Orbigny, 1846), lateral view, lower Sarmatian, sample HA 573/2; 8. *Anomalinoides dividens* Łuczkowska, 1967, a: spiral view, b: umbilical view, lower Sarmatian, sample HA 573/2; 9. *Elphidium crispum* (Linnaeus, 1758), lateral view, upper Badenian, sample HA 66/21; 10. *Elphidium* cf. *subumbilicatum* (Czjzek, 1848), lateral view, lower Sarmatian, sample HA 66/6; 11. *Elphidium* cf. *grilli* Papp, 1963, lateral view, lower Sarmatian, sample HA 66/6; 12. *Elphidium* cf. *koberi* Tollmann, 1955, a: lateral view, b: oblique apertural view, lower Sarmatian, sample HA 66/6

Planktic foraminifera occur in very small quantities in most samples (Fig. 9), but they are badly preserved and tiny. In a few samples *Orbulina suturalis* was detected (HA 66/17, HA 511/5, HA 540/6, HA 573/13, /8, /7) (Fig. 10.5).

In respect to stratigraphy the most important regional marker found in several samples of HA 66 was *Pappina neudorfensis* (Fig. 10.4) which is considered to indicate *Bolivina-Bulimina* Zone (Haunold, 1995) and a late Badenian age. Individuals of *Bolivina* and *Bulimina* are recorded in several samples (see above) pointing also at the *Bulimina-Bolivina* Zone but are not considered very strong markers. A remarkable record is the nearly monospecific occurrence of *Saccammina sarmatica* in sample HA 573/3 (Fig. 10.6a-c) and the very abundant occurrence of *Anomalinoides dividens* (Fig. 10.8a, b) and *Elphidium aculeatum* (Fig. 10.7) in sample HA 573/2. The later are markers for early Sarmatian and *S. sarmatica* is restricted to the Sarmatian. In the top sample HA 66/7 the fauna consists exclusively of *Elphidium crispum* (Fig. 10.9) but in HA 66/6 *E.* cf. *subumbilicatum* (Fig. 10.10), *E.* cf. *grilli* (Fig. 10.11), and *Elphidium* cf. *koberi* (Fig. 10.12a, b) occur portending a Sarmatian age (Cicha et al., 1998; Schütz et al., 2007).

### Calcareous nannoplankton

**Fig. 11:**
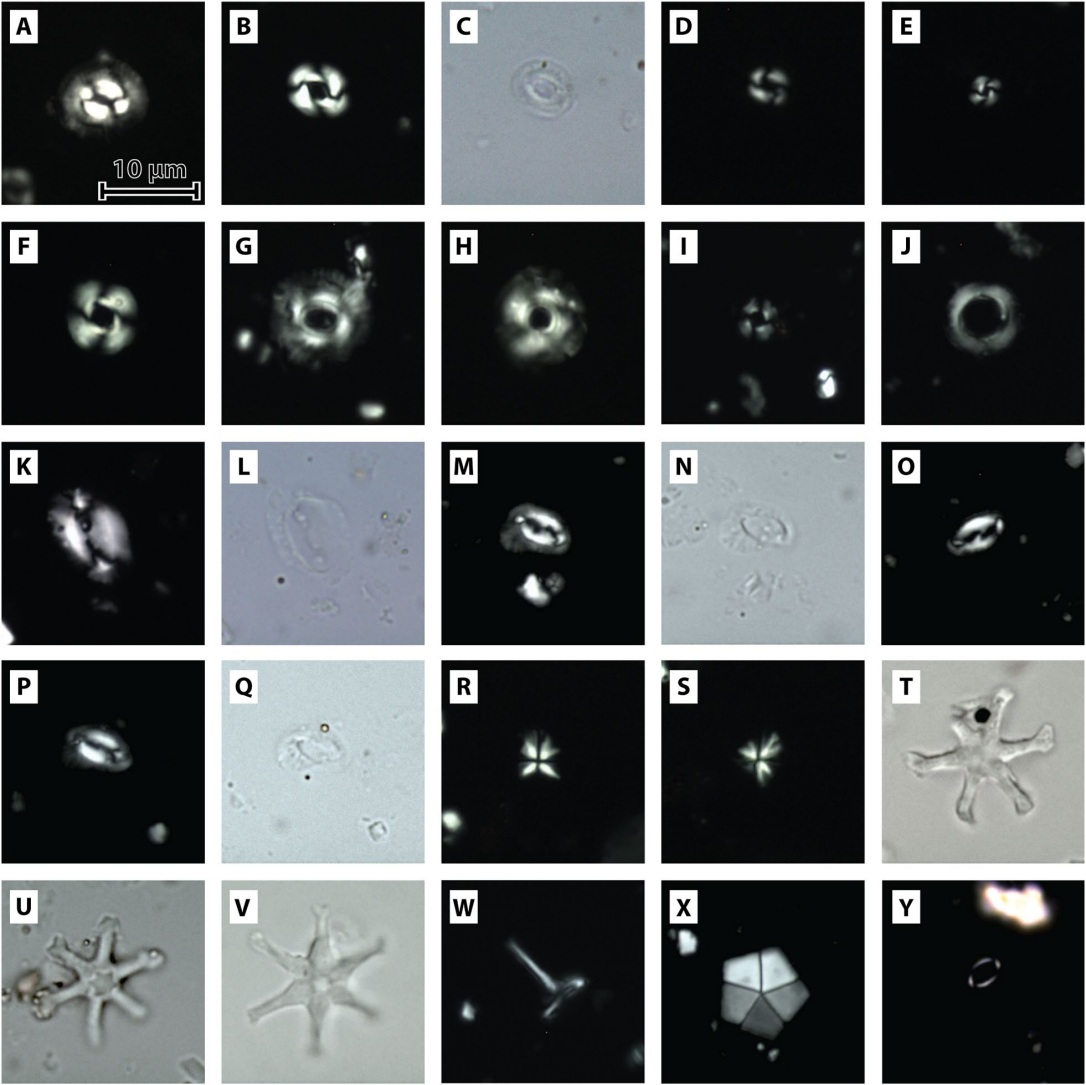
Microphotographs of calcareous nannofossils taxa found in the investigated samples. Taxa are shown under crossed and parallel nicols as specified and presented as equally sized images to illustrate size differences. **A** *Coccolithus pelagicus* subsp. *pelagicus* (Wallich, 1877) Schiller (1930), crossed nicols, sample: HA 511/11; **B** *Reticulofenestra pseudoumbilicus* (Gartner, 1967) Gartner (1969), crossed nicols, sample: HA 511/08; **C** *Reticulofenestra pseudoumbilicus* (Gartner, 1967) Gartner (1969), parallel nicols, same specimen as **B**; **D** *Reticulofenestra haqii* Backman (1978), crossed nicols, Sample: HA 573/07; **E** *Reticulofenestra minuta* Roth (1970), crossed nicols, sample: HA 573/07; **F** *Cyclicargolithus floridanus* (Roth & Hay, in Hay et al., 1967) Bukry (1971), crossed nicols, sample: HA 573/07; **G** *Calcidiscus premacintyrei* Theodoridis (1984), sample: HA 511/08; **H** *Calcidiscus macintyrei* (Bukry and Bramlette, 1969) Loeblich and Tappan (1978), crossed nicols, sample HA 511/11; **I** *Umbilicosphaera jafari* Muller (1974), crossed nicols, sample HA 511/11; **J** *Umbilicosphaera rotula* (Kamptner, 1956) Varol (1982), crossed nicols, sample HA 511/08; **K** *Helicosphaera carteri* (Wallich, 1877) Kamptner (1954), crossed nicols, sample HA 66/08; **L** *Helicosphaera carteri* (Wallich, 1877) Kamptner (1954), parallel nicols, same specimen as **K**; **M** *Helicosphaera vedderi* Bukry (1981), crossed nicols, sample HA 66/08; **N** *Helicosphaera vedderi*, parallel nicols, sampe specimen as **M**; **O** *Helicosphaera stalis* Theodoridis (1984), crossed nicols, sample HA 66/08; **P** *Helicosphaera walbersdorfensis* Müller (1974), crossed nicols, sample HA 66/08; **Q** *H. walbersdorfensis*, parallel nicols, same specimen as **P**; **R** *Sphenolithus moriformis* (Brönnimann & Stradner, 1960) Bramlette & Wilcoxon (1967), crossed nicols 90°, sample HA 511/08; **S** *S. moriformis*, crossed nicols 45°, same specimen as **R**; **T** *Discoaster* sp. Tan Sin Hok (1927) (cf. *D. exilis*; Martini and Bramlette, 1963), parallel nicols, sample HA 511/08; **U** *Discoaster exilis* Martini and Bramlette (1963) (cf. *D. cauliflorus* Browning & Bergen, in Browning et al., 2017), parallel nicols, sample HA 573/07; **V** *Discoaster exilis* Martini and Bramlette (1963), parallel nicols, sample HA 66/19; **W** *Rhabdosphaera xiphos* (Deflandre & Fert, 1954) Norris (1984), crossed nicols, sample HA 511/11; **X** *Braarudosphaera bigelowii* (Gran & Braarud, 1935) Deflandre (1947), crossed nicols, sample HA 66/07; **Y** *Syracosphaera* sp. (cf. *S. mediterranea*) Lohmann (1902), crossed nicols, sample HA 573/02

Calcareous nannoplankton has been studied in boreholes HA 66, HA 511, and HA 573 (Fig. 11). Samples HA 66/30 and HA 66/28 are barren in nannoplankton, but HA 66/19 shows a diverse and moderately preserved flora containing *Discoaster exilis*, *Coccolithus pelagicus*, *Reticulofenestra pseudoumbilicus* (> 7 μm), common *Calcidiscus premacintyrei*, *Helicosphaera walbersdorfensis*, rare *Cyclicargolithus floridanus*, and *Coronocyclus nitescens*. Samples HA 66/08 and HA 66/07 were both moderately to well preserved with common occurrences of *H. walbersdorfensis*, rare *H. stalis* and *H. vederi*, abundant *C. pelagicus* and *R. pseudoumbilicus*, the occasional presence of *C. floridanus*, but the notable absence of *C. premacintyrei*. Reworking of Cretaceous and Paleogene taxa is very common. Sample HA 66/5 is nearly barren in stratigraphically indicative autochthonous taxa, although common *C. pelagicus* and rare *Reticulofenestra minuta*, *R. haqii*, and *C. floridanus* occur; reworked Cretaceous and Paleogene taxa are common.

Of the 11 samples analyzed from Borehole HA 573, nine were nearly barren in autochthonous nannofossil assemblages. Samples HA 573/19, /17, /14, /13, /11, /09, /08, /04, and /03, however, contain a diverse and surprisingly well preserved and diverse allochthonous assemblage. The assemblage contains nannofossils of Early/Late Cretaceous (Aptian to Maastrichtian) to Paleogene (early Paleocene to late Oligocene) age. Rare, potentially autochthonous taxa include *Coccolithus pelagicus*, *Cyclicargolithus floridanus,* and *Reticulofenestra minuta*, although their long stratigraphic range may result in a significant contribution of allochthonous specimens to these taxa. However, rare occurrences of *R. pseudoumbilicus* and *Sphenolithus moriformis* indicate at least occurrences of autochthonous nannoplankton in these samples. Sample HA 573/02 shows nearly no reworking and a dominance of small placoliths assigned to the taxon *Syracosphaera* sp. (cf. *S. mediterranea*), accompanied by common *C. pelagicus*, as well as rare medium to small reticulofenestrids and *Umbellosphaera* sp.

From Borehole HA 511, nannofossil assemblages were investigated in 3 samples (HA 511/11, /08, /04). All three samples show well-preserved nannofossil assemblages with very low reworking (generally well below 1% of the total assemblage). Quantitative analysis of these well-preserved samples was carried out. Sample HA 511/11 shows high occurrences of *Umbilicosphaera jafari* (~ 49.5%), with common occurrences of *R. pseudoumbilicus* (~ 11.6%), *R. haqii* (~ 9.3%), *R. minuta* (10.0%), and *C. pelagicus* (~ 7.2%). Accessory species include *Calcidiscus leptoporus* subsp. small (< 5 µm; ~ 3%), *Umbilicosphaera rotula* (~ 2.8%), *Syracosphaera* sp. (~ 2.1%), *Helicosphaera carteri* (~ 1.5%), and *S. moriformis* (~ 1.2%). Sample HA 511/8 shows a decrease in *U. jafari* (~ 25.2%) and an increase in *R. haqii* (~ 26.9%) and *R. pseudoumbilicus* (~ 19.8%). *C. pelagicus* (12.6%) and *R. minuta* (~ 7.1%) are also common. Abundances of *Syracosphaera* sp. (~ 1%) are somewhat lower. Other taxa are *H. carteri* (~ 1.5%) and *S. moriformis* (~ 1.4%). Rare occurrences of reworked Paleogene *Reticulofenestra bisecta* (~ 1.3%) and *Reticulofenestra dictyoda* (~ 0.8%) are also noteworthy in sample HA 511/8, as otherwise, reworking is very rare in borehole HA 511. Sample HA 511/4 shows the highest abundances of *R. minuta* (~ 32.1%), with common *U. jafari* (~ 31.2%), and reduced *R. haqii* (~ 11.2%). Other taxa include *C. pelagicus* (~ 6.5%), *Syracosphaera* sp. (~ 3.4%), and *H. carteri* (~ 1%).

### Ostracodes

**Fig. 12:**
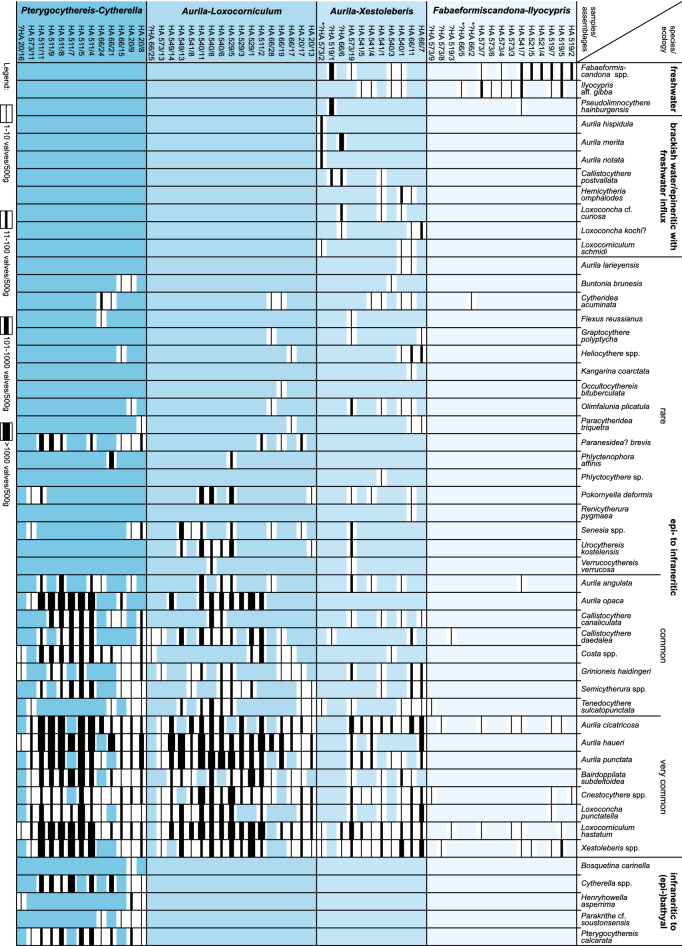
Occurrence and frequency of ostracode taxa (modified after Gross, 2002; Sarmatian samples marked with an asterisk; barren samples not displayed)

For ostracodes, 66 samples from all boreholes (except core HA 518; see 5.1) were investigated. Ten samples were entirely barren (Fig. 12; Gross, 2002, 2006).

**Fig. 13:**
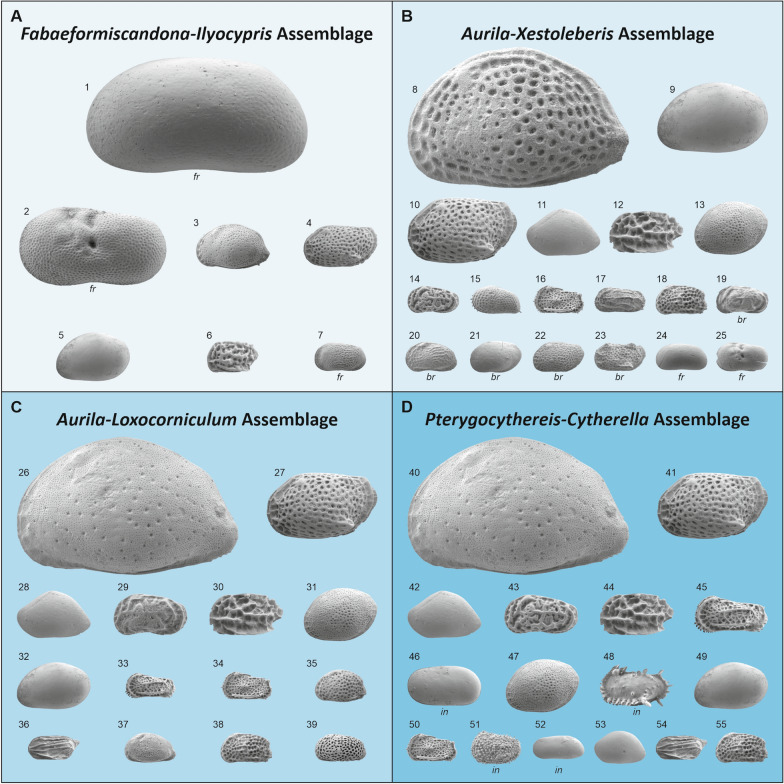
Schematic illustration of ostracod assemblages (not to scale). **A** *Fabaeformiscandona*-*Ilyocypris* assemblage, **B** *Aurila*-*Xestoleberis* assemblage, **C** *Aurila*-*Loxocorniculum* assemblage, **D** *Pterygocythereis*-*Cytherella* assemblage. Size of figured valves refer to their frequency in the assemblage. Paleoecologically significant taxa marked with *fr* (freshwater), *br* (brackish water or epineritic with freshwater influx) and *in* (infraneritic to (epi-)bathyal). 1: *Fabaeformiscandona* sp. 1; 2: *Ilyocypris* aff. *gibba*; 3: *Aurila cicatricosa*; 4: *Loxocorniculum hastatum*; 5: *Xestoleberis tumida*; 6: *Cnestocythere truncata*; 7: *Pseudolimnocythere hainburgensis*; 8: *Aurila punctata*; 9: = 5; 10: = 4; 11: *Bairdoppilata subdeltoidea*; 12: *Cnestocythere lamellicosta*; 13: *Loxoconcha punctatella*; 14: *Callistocythere canaliculata*; 15: *Cytheridea acuminata*; 16: *Grinioneis haidingeri*; 17: *Olimfalunia plicatula*; 18: *Tenedocythere sulcatopunctata*; 19: *Callistocythere postvallata*; 20: *Hemicytheria omphalodes*; 21: *Loxoconcha* cf. *curiosa*; 22: *Loxoconcha kochi*?; 23: *Loxocorniculum schmidi*; 24: = 1; 25: = 2; 26: *Aurila haueri*; 27: = 4; 28: = 11; 29: *Callistocythere daedalea*; 30: = 12; 31: = 13; 32: = 5; 33: *Costa punctatissima*; 34: = 16; 35: *Pokornyella deformis*; 36: *Semicytherura galea*; 37: *Senesia trigonella*; 38: = 18; 39: *Urocythereis kostelensis*; 40: = 26; 41: = 4; 42: = 11; 43: = 14; 44: = 12; 45: = 33; 46: *Cytherella* aff. *compressa*; 47: = 13; 48: *Pterygocythereis calcarata*; 49: = 5; 50: = 16; 51: *Henryhowella asperrima*; 52: *Parakrithe* cf. *soustonensis*; 53: *Paranesidea*? *brevis*; 54: = 36; 55: = 18

The samples were grouped into four assemblages based on the occurrence of paleoecologically distinct taxa (Figs. 12, 13); for details to the assumed paleoecology of ostracod taxa see Gross (2002); bathymetric terms follow Liebau (1980).

- *Fabaeformiscandona*-*Ilyocypris* assemblage: this assemblage comprises samples with a low number of specimens and species. The thin-shelled freshwater ostracodes *Fabaeformiscandona* spp. (almost exclusively juvenile valves) and *Ilyocypris* aff. *gibba* (only adult valves) dominate the ostracod fauna. One sample contained the freshwater taxon *Pseudolimnocythere hainburgensis*. Epi- to infraneritic elements (in particular *Aurila* spp., *Cnestocythere* spp., *Loxocorniculum hastatum*, *Xestoleberis* spp.) occur subordinately. Freshwater indicators are missing from samples HA 519/3, HA 573/8 and HA 573/9, which yielded only very few individuals. They are placed with reservation as well as the lower Sarmatian samples HA 66/2 and HA 66/5 due to their position in the cores in this assemblage.
- *Aurila*-*Xestoleberis* assemblage: compared to the *Fabaeformiscandona*-*Ilyocypris* assemblage ostracode abundance and diversity is higher. *Aurila* spp., *Xestoleberis* spp., and *Loxocorniculum hastatum* prevail the ostracode fauna. Other epi- to infraneritic forms (especially *Bairdoppilata subdeltoidea*, *Callistocythere canaliculata*, *Cnestocythere* spp., *Cytheridea acuminata*, *Grinioneis haidingeri*, *Loxoconcha punctatella*, *Olimfalunia plicatula*, *Tenedocythere sulcatopunctata*) occur subordinately. In most samples, elements characteristic for brackish or epineritic, freshwater influenced environments are documented (*Callistocythere postvallata*, *Hemicytheria omphalodes*, *Loxoconcha* cf. *curiosa*, *Loxoconcha kochi*? and *Loxocorniculum schmidi*) as well as freshwater species (*Fabaeformiscandona* spp., *Ilyocypris* aff. *gibba*). Although sample HA 519/1 is highly dominated by *Fabaeformiscandona* cf. *pokornyi* and *Pseudolimnocythere hainburgensis* (c. 95%), this sample has been tentatively placed in the *Aurila*-*Xestoleberis* assemblage due to the occurrence of the brackish water form *Callistocythere postvallata*. The lower Sarmatian samples HA 66/6 and HA 573/2 are provisionally assigned to this assemblage due to the poor Sarmatian fauna.
- *Aurila*-*Loxocorniculum* assemblage: samples of this assemblage differ from the *Aurila*-*Xestoleberis* assemblage by much more numerous ostracodes and the predominance of *Aurila* spp. and *Loxocorniculum hastatum*. Frequently, *Bairdoppilata subdeltoidea*, *Callistocythere daedalea*, *Cnestocythere* spp., *Loxoconcha punctatella*, and *Xestoleberis* spp. co-occur; the latter being very abundant in some samples (e.g., HA 529/1). Fresh- and brackish water elements are completely absent. *Aurila opaca* and, rarely, *Occultocythereis bituberculata*, *Paranesidea*? *brevis*, and *Phlyctenophora affinis* appear in this community. The assignment of sample HA 66/25 remains questionable due to the rare material (3 valves).
- *Pterygocythereis*-*Cytherella* assemblage: this assemblage is rich in species and individuals and differs from the *Aurila*-*Loxocorniculum* assemblage by the frequent occurrence of *Pterygocythereis calcarata* and *Cytherella* spp. Sample HA 20/16 yielded only very few valves, however, due to the occurrence of *Henryhowella asperrima* it is assigned to this assemblage.

## Discussion

### Stratigraphy

Already Wessely (1961) assigned the studied Badenian sediments in the Hainburg Mountains to the *Bulimina-Bolivina* Zone and therefore into the upper Badenian. Further support for this assignment came from the study by Haunold (1995) who stated that *Pappina neudorfensis* is indicative for the *Bulimina-Bolivina* Zone (cf. Cicha et al., 1998). In the herein studied material *P. neudorfensis* was also found supporting this assignment. The occurrence of *Anomalinoides dividens* and *Elphidium aculeatum* in the top of HA 573 clearly indicates an early Sarmatian age. However, the dominating taxon *Saccammina sarmatica* in HA 573/3 already points at a Sarmatian age below. In HA 66/6, *E.* cf. *koberi, E.* cf. *grilli* and *E.* cf. *subumbilicatum* proof Sarmatian age. In both boreholes also ostracodes indicate a late Badenian age for most of the successions (Danielopol et al., 1991) but also for the Sarmatian in the top of HA 66 and HA 573 (Gross, 2002, 2006). For HA 573 ostracodes give a Sarmatian age for sample HA 573/2 but foraminifera already assign sample HA 573/3 to the Sarmatian which is c. 6 m below.

Due to ecological effects and extending knowledge about the total stratigraphic range of ‘index’ ostracodes, earlier proposed ostracod zonations (e.g., Jiricek & Riha, 1991) are of limited value (Gross, 2006; see also Hajek-Tadesse & Prtoljan, 2011 and Seko et al., 2012). Nonetheless, the found ostracod taxa clearly indicate a Badenian age for most of the samples, which could be refined to a late Badenian age by foraminifera. Ostracods specifying an early Sarmatian age have been found only in the uppermost part of cores HA 66 and HA 573 suggesting a late Badenian age for the deposits below (Gross, 2006; see above).

The calcareous nannoplankton assemblage indicates nannoplankton zone (lower) NN6 (Martini, 1971), MNN6b (Fornaciari et al., 1996) and CNM8 (Backman et al., 2012) through the presence of *Cyclicargolithus floridanus* and *Calcidiscus premacintyrei* together with the absence of *Sphenolithus heteromorphus*. Samples HA 66/08 and HA 66/07 were assigned to (upper) NN6 corresponding to (lower) MNN7 and lower CNM9, through the co-occurrence of common *Helicosphaera vedderi* and *H. walbersdorfensis*, with rare *H. stalis* and *R. pseudoumbilicus,* as well as the absence of *C. premacintyrei* (Backman et al., 2012; Fornaciari et al., 1996; Martini, 1971). NN6 gives an unambiguous very clear indication of Serravallian, CNM8 indicates lower Serravallian, CNM9 upper Serravallian. For the Central Paratethys CNM8 correlates with the upper Badenian, CNM 9 would indicate Sarmatian. The latter, however, is primarily based in our material on the absence of *C. premacintyrei* without the expected occurrence of *C. macintyrei* (> 11 µm; Fornaciari et al., 1996) and, therefore, not a very strong argument.

The first occurrence of the dinocyst *Unipontidium aquaeductum* marks the base of the dinoflagellate zone DM5 after King (2016) and Gradstein et al. (2020) and its last occurrence the top of DM5 ranging from upper Langhian to middle Serravallian. *Habibacysta tectata* which occurs in samples HA 66/19 and HA 66/21 (Soliman et al., 2022) has its first occurrence in the lowermost Serravallian. Both taxa together clearly indicate a late Badenian age. *Cannosphaeropsis passio* represented in samples HA 66/17 and HA 66/19 (Soliman et al., 2022) is considered a marker for dinocyst zone DM6b with its first occurrence at the base and its last occurrence at the top of DM6b. Following the DM zonation this taxon indicates upper Serravallian (Gradstein et al., 2020) which fits with the Sarmatian of the Central Paratethys. One drawback, however, is the co-occurrence of *C. passio* and *U. aquaeductum* in sample HA 66/19 requiring a re-evaluation of the dinocyst biozonation (Soliman et al., 2022).

All biostratigraphic data support a late Badenian and add for the topmost parts an early Sarmatian age. In respect to the established sequence stratigraphy the studied Badenian material belongs to the regional sequence Ba3 (Kranner et al., 2021a; Siedl et al., 2020; Strauss et al., 2006). Ba3 can be correlated with the 3^rd^ order sequence TB 2.5 of Hardenbol et al. (1998) which is bound by the lowstands Ser2 and Ser3. This sequence stratigraphic approach has been verified both in the southern (Strauss et al., 2006) and the northern Vienna Basin (Siedl et al., 2020). The studied cores from Bad Deutsch-Altenburg start with a transgression which can be correlated to the TST following lowstand Ba3 (Ser2) and is terminated within the highstand of Ba3 (TB 2.5) (Fig. 1). The following lower Sarmatian sediments can be correlated to the regional sequence Sa1 (Kranner et al., 2021a; Siedl et al., 2020; Strauss et al., 2006) corresponding to TB 2.6 of Hardenbol et al. (1998) (Fig. 1). The boundary between the upper Badenian and the lower Sarmatian is recognized by foraminifera and ostracodes in the uppermost parts of several boreholes, but a hiatus which should be expected (Kranner et al., 2021a) was macroscopically not detected. This can possibly be explained by the widely uniform marls at the top of the Badenian and bottom of the Sarmatian. However, even the exact boundary cannot be defined (see foraminifera and ostracodes above).

Lithostratigraphically, the studied upper Badenian sediments have to be classified with the Rabensburg Formation defined and described by Harzhauser et al. (2020) (Fig. 1). This unit belongs to the Baden Group, is well known from many deep wells in the Vienna Basin and reaches up to 1000 m in thickness being laterally highly variable due to erosion at the Badenian/Sarmatian boundary. Lithology is highly variable with a dominance of marls but also with corallinacean limestones, fluvial gravel and even lignite seams (Harzhauser et al., 2020). The corallinacean limestones resemble that of the recently defined Leitha Formation which is, however, restricted to the middle Badenian (Harzhauser et al., 2020). In the Slovak part of the Vienna Basin these sediments of the upper Badenian are assigned to the Studienka Fm. (e.g., Kováčová & Hudáčková, 2009). This formation is, however, not formally defined. The lower Sarmatian sediment can be assigned to the Holíč Formation which has been first described by Elečko and Vass (2001) in the Slovak part of the Vienna Basin (cf. Harzhauser et al., 2020).

A similar shallow water setting of upper Badenian—lower Sarmatian sediments occurs close by at the Devínska Kobyla Hill in the vicinity of Devínska Nová Ves a NW district of the city of Bratislava on the territory of the Slovak Republic. This location is situated c. 8 km NE of the study area in Bad Deutsch-Altenburg and belongs geologically to the Malé Karpaty Mts. (Little Carpathians) (Fig. 2). There are sediments exposed which have been studied, e.g., by Barath et al. (1994), Hyžný et al. (2012) and Bitner et al. (2014). The upper Badenian sediments are classified with the Studienka Formation which includes the Sandberg Member, the lower Sarmatian part belongs to the Holíč Fm. and includes the Radimov Mb. The Studienka Fm. is a basinal facies which consists mostly of calcareous clay and claystone, the Sandberg Member represents its marginal environment. This member starts with transgressive breccias and conglomerates on Mesozoic rocks overlain by sands, gravel, silts, and bioclastic limestones; overall siliciclastics dominate. The Sandberg Mb. reaches a thickness of 100 m and correlates with the studied successions in Bad Deutsch-Altenburg but the dominant siliciclastics with coarse breccias of crystalline rock material reflect the local crystalline basement at the type locality. The Studienka Fm. can be considered an equivalent of the Rabensburg Formation in the Austrian upper Badenian part of the Vienna Basin sediments. The Studienka Fm., however, is not correctly defined and formalized and the Rabensburg Fm. is considered to have priority (Harzhauser et al., 2020). The upper part of the Sandberg Mb. (Sandberg facies 4) has been dated into NN6. An exact boundary between Badenian and Sarmatian has not been defined so far (Hyžný et al., 2012).

### Sediment distribution pattern

**Fig. 14:**
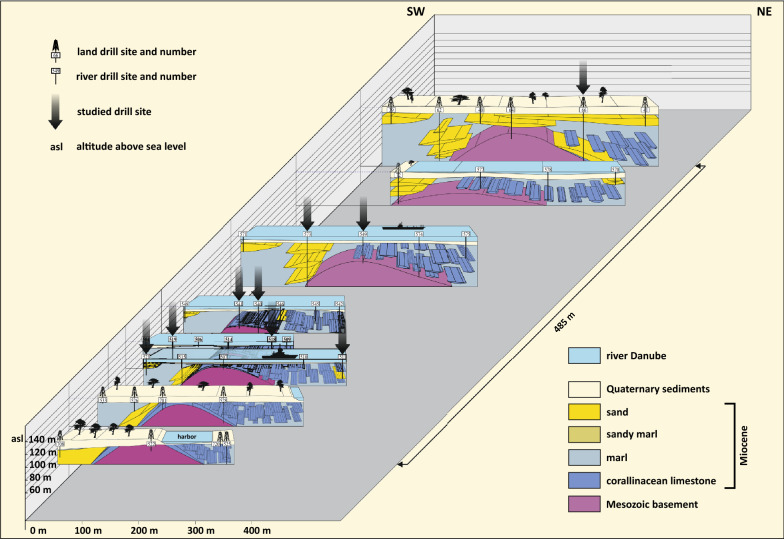
Schematic reconstruction of the sedimentary environment based on all indicated land and river drill sites. On top of the ridge formed by Mesozoic basement rocks a thin cover of corallinacean limestone is indicated in the SE. The high amount of corallinacean limestone NE of the ridge and coarser siliciclastics in the SW is schematically visualized as discontinuous bodies embedded mostly in marl

During the late Badenian the sediment distribution in the studied area was determined by paleotopography, in particular, by the existence of the Mesozoic ridge which acted as a strong physical barrier (Fig. 14). The surface of the ridge is covered by a blanket of carbonate rocks which are overlain in the SW by coarser siliciclastics but grade into a thick carbonate dominated sequence in the NE. This and the data of fossil assemblages (see chapter 6.3 and 6.4) support the idea that in the west of the ridge rivers or creeks existed which fluvially delivered quartz sand and freshwater biota. The ridge acted as barrier prohibiting transport of the sandy sediment to the east allowing the development of a terrigenously influenced carbonate factory (Fig. 14). This factory was active as long as water depth was shallow enough for carbonate production but was terminated upsection and covered by predominantly marl sediment which represents the typical basinal sediments in the Vienna Basin locally called “Tegel” or “Baden Tegel”.

### Taphonomic and diagenetic signals

Calcareous nannoplankton and dinoflagellate cyst assemblages show generally a strong mixture of autochthonous and allochthonous elements which can relatively easy be identified when the mixture of elements derive from differently aged sources. In both biota groups a high content of Upper Cretaceous and Paleogene elements is present (heterochronous taphocoenoses) (see also Soliman et al., 2022). These can be derived from different sources:

(i) Sediments of the Gosau Group on top of the Northern Calcareous Alps and of the Rhenodanubian Flysch Zone are restricted to this time interval with termination of sedimentation in the Eocene. Both geotectonic units became uplifted in the course of the Alpine orogeny during the Oligocene to Early Miocene, became exhumed and exposed to erosion (Trautwein et al., 2001). Both units crop out at the western margin of the Vienna Basin and the eroded material could have easily been transported in suspension across the basin to its eastern margin.
(ii) A similar geotectonic history is reported from the Western Carpathians (Kováč et al., 1994) which is very close by and could also have acted as source area.
(iii) An additional source for Paleogene reworked biota is the occurrence of the upper Eocene sediments of Wimpassing an der Leitha (Burgenland) which represents an erosional relict of a former widespread Eocene cover (Fuchs, 1980; Pahr & Herrmann, 2000). These sediments are currently very poorly exposed (and preserved) but consist of limestones and sandstones containing coralline algae, larger foraminifers (*Nummulites*), bryozoan, molluscs etc. (Zorn, 2000).

Concerning calcareous nannoplankton, all samples from borehole HA 573 are dominated by moderately to well preserved reworked nannofossils. One sample (HA 573/07) shows rare occurrences of potentially autochthonous taxa such as *Helicosphaera carteri* and *Sphenolithus heteromorphus* (one specimen). Only the lower Sarmatian sample HA 573/02 shows rare, reworked material and a dominance of small placoliths assigned to *Syracosphaera* spp. While this is generally considered a taxon reflective of oligotrophic conditions, blooms of *Syracosphaera halldalii* were recently recorded in estuarine environments (Skejić et al., 2021). Together with rare occurrences of *R. minuta*, *R. haqii*, *C. pelagicus*, and *Braarudosphaera bigelowii*, we thus interpret the early Sarmatian assemblage of HA 573/02 to reflect estuarine conditions, with low terrigenous influx, that likely exhibited variable salinity and nutrient levels. In sum, borehole HA 573 reflects a sequence dominated by high turbidity riverine influx leading to a complete absence of autochthonous taxa or the presence of specialized low salinity assemblages (in the Sarmatian sample) in times of lower turbidity and thus terrigenous influx.

On the contrary, the three samples investigated from borehole HA 511 show abundant but variable occurrences of *Umbilicosphaera jafari*, *Reticulofenestra minuta,* and *R. pseudoumbilicus*, and common *C. pelagicus*, but very low amounts of allochthonous taxa.

Ostracodes show a different composition of autochthonous and allochthonous assemblages as calcareous nannoplankton and dinoflagellates. They represent a mixture of marine – autochthonous – and freshwater – allochthonous – elements which are probably all of late Badenian age (synchronous taphocoenoses). The freshwater ostracodes are particularly abundant in the boreholes SW of the Mesozoic ridge (HA 514, HA 519, HA 521) which indicate fluvial transport from the south (Danielopol et al., 1991; Gross, 2002, 2006). This indicates that these ostracodes were contemporaneously transported into the marine environment together with characean oogonia (Danielopol et al., 1991). Their predominant occurrence in the SW of the Mesozoic ridge again indicates that this ridge acted as a barrier; coarser siliciclastics and transported freshwater ostracodes were retained in the west.

Overall, a separation occurs along the Mesozoic ridge between sediments with high amounts of allochthonous biotic elements in the SW of the ridge and mostly autochthonous elements NE of the ridge. Whereas calcareous nannoplankton and dinocysts show a mixture of Upper Cretaceous, Paleogene and Miocene assemblages ostracodes contain some synsedimentary allochthonous elements (synchronous taphocoenoses) which were fluvially transported into the shallow marine environment SW of the ridge.

Another important feature of the region is karstification which was also a major concern in the Hainburg powerplant project because of the hazard for the mineral springs (hot iodine–sulphur springs; Gangl, 1990). Both, the Triassic dolomite and the Badenian corallinacean limestones are strongly karstified and karstification occurs also in the impure, marly to sandy, corallinacean limestones (Gangl, 1988, 1990). This karstification can be observed on the land surface but it was repeatedly discovered in the subsurface during drilling. The karstification of the Triassic carbonate rocks started already in pre-Miocene times because the upper Badenian sediment occurs as infillings in karst cavities of the Triassic dolomite and the surface of these karst features is bored by marine biota. Both karst systems, however, are nowadays interconnected and act as continuous karst aquifer. This intense karstification may be one reason for the poor and selective preservation of foraminifera possibly in relation with the mineralized water of the spa.

### Paleoenvironment and paleoclimate

#### Water depth

The geological situation in the studied area at Bad Deutsch-Altenburg reflects a marine transgression. Due to variable positions of the transgression horizons in the various boreholes the transgression can be traced from lower to higher (actual) altitude levels. The lowermost detected level is documented with a transgression conglomerate in borehole HA 66 which occurs at around 50.9 m above actual sea level (asl). In borehole HA 540 the transgression horizon is at around 102.3 m asl. When at the position of HA 540 the Mesozoic basement was flooded there was a difference of about 50 m to HA 66. However, further south of the studied drillings (e.g., at the Kirchenberg and the Pfaffenberg in Bad Deutsch-Altenburg) the transgressional contact of the upper Badenian is much higher in altitude. This much wider range can only be explained by local/regional tectonic movements what has been already suspected for other areas in the Vienna Basin (Kranner et al., 2021a). Wessely (1961) expected various tectonic structures in the Hainburg Mountains and Gangl (1988, 1990) found good evidence for faults in the area for the planned powerplant project Hainburg, in particular on the western side of the Mesozoic ridge. The Neogene sediments in the actual studied cores do not show major disturbances which would point to tectonic activities such as tilting and displacements along faults and we can consider the difference in altitude may reflect depositional depth. This means that at the onset of the transgression in HA 540 water depth in HA 66 was around 50 m maximum at this time (by neglecting contemporaneous sedimentation).

For reconstructing water depth LBF are widely used proxies. In our material, *Borelis melo* is nearly restricted to basal samples directly above the transgression horizon. This indicates a very shallow water depth and the breccias/conglomerates point at higher water energy levels than most other sediments in the studied material. *B. melo* is the only representative of the alveolinids in the Miocene of the Central Paratethys. Alveolinids are also represented monospecifically in the Red Sea today, where *Borelis schlumbergeri* is the only taxon. Reiss and Gvirtzman (1966) reported the depth distribution of this species between 1.5 and 20 m in the Gulf of Elat/Aqaba. Hottinger (1977) and Reiss and Hottinger (1984) mentioned its main distribution between 25 and 35 m, occurring in lower percentages between 10 and 60 m and become rare in shallower and deeper sites. From the Bay of Safaga (Red Sea) Haunold et al. (1997, 1998) described the taxon typical for the *Quinqueloculina mosharrafai-Borelis schlumbergeri-Brizalina simpsoni* Association from 23 to 33 m. In the Red Sea (and Gulf of Aqaba) the test shape varies widely from oval to fusiform. The herein studied upper Badenian specimens are all spherical without any fusiform individuals. Although the depth range is very wide the substrate seems to be predominantly hard bottom both in the Red Sea and the late Badenian of Bad Deutsch-Altenburg. Their restriction to the basal transgression horizon may be caused by possible shelter between the coarser components and high suspension load in the water column, reducing light availability.

The most obvious and dominating LBF taxon in the studied samples is *Amphistegina mammilla* which can reach rock-forming quantities, so-called *Amphistegina* marl. It occurs in a wide range of lithologies, from limestones to marls, but prefers a terrigenously influenced/dominated carbonatic sediment (sandy limestone, sandy marl, marly limestone, and rarely marly sand) (see above). Because of this preference it is overall rare in borehole HA 573 because of the high sand share in the sediments. It also vanishes above HA 66/18 when the sediments become too pelitic. Depth distribution of modern *Amphistegina* species is very wide ranging from 0 m down to > 150 m, their distribution, however, is species dependent. Comparable morphologies exist between fossil *A. mammilla* and extant *A. lessonii* and *A. radiata*. Both recent taxa occur between 0 and 70/90 m with an acme between 20 to 30 m as reported from the NW Pacific in clear ocean waters (Hohenegger, 1995, 2000; Hohenegger et al., 1999). A simple correlation between test shape and depth has been proposed by Hallock (1979, 1981), Hallock and Glenn (1986), and Hallock et al. (1986) showing thicker tests in shallow water and flatter test shapes with increasing depth. In the herein studied material (11 samples, 2552 individuals, Table 2) no such trend was observed and no depth/test shape relationship can be derived. Overall, the tests are relatively flat, and this may point to a low transparency due to high suspension load, which agrees with the sediment types in which *Amphistegina* occurs. This low transparency through the sections may overprint possible depth/light dependencies. Renema (2005) also drew a similar conclusion on modern *Amphistegina* occurrences offshore Indonesia.

The third representative of LBF is *Planostegina* sp. This taxon was only recorded in limestone facies and could be studied only in thin sections. Two taxa are reported from the upper Badenian (Cicha et al., 1998) but an identification at the species level was not possible. Modern *Planostegina* taxa are predominantly known from low light environments in the deeper photic zone (e.g., Hohenegger, 2004). This fits well with the occurrence in the BDA cores where *Planostegina* occurs mainly in samples from greater water depth (HA 66/23, HA 518/17).

Although coralline red algae are known to exhibit a distinct depth distribution in their taxonomic composition (e.g., Aguirre et al., 2017) in the studied material no such distributional pattern has been detected. This is similar to the negative result in larger benthic foraminifers.

Four ostracode assemblages could be differentiated (Figs. 12, 13): the *Fabaeformiscandona-Ilyocypris* assemblage (FI), the *Aurila-Xestoleberis* assemblage (AX), the *Aurila-Loxocorniculum* assemblage (AL) and the *Pterygocythereis-Cytherella* assemblage (PC; see 5.5). The ostracode association of FI suggests an epineritic paleoenvironment with strong terrigenous input indicated by allochthonous freshwater ostracodes (*Fabaeformiscandona* spp., *Ilyocypris* aff. *gibba* and – one sample – *Pseudolimnocythere hainburgensis*). In part, the presence of *Xestoleberis* spp. indicates the proximity of phytal areas (e.g., seagrass meadows). Equally, assemblage AX signals an epineritic setting with some terrigenous/freshwater influx due to the occurrence of displaced continental (*Fabaeformiscandona* spp., *I*. aff. *gibba*, *P*. *hainburgensis*) and ‘brackish water’ ostracodes (*Callistocythere postvallata*, *Hemicytheria omphalodes*, *Loxoconcha* cf. *curiosa* and *Loxocorniculum schmidi*). The common occurrence of *Xestoleberis* spp. points to densely vegetated areas. The ostracodes recorded in assemblage AL indicate a fully marine, epineritic, mostly sandy, vegetated paleoenvironment. The faunas provide no hints at any freshwater influx. For the PC assemblage a fully marine habitat at the transition from the epineritic to the infraneritic zone can be suggested. *Pterygocythereis* clearly points to a deposition below the storm wave base (infraneritic) but still within the euphotic (lower infralittoral) zone due to the presence of *Xestoleberis*. For the documented cytherellids and *Bosquetina carinella*, *Henryhowella asperrima*, and *Parakrithe* cf. *soustonsensis* a preference for infraneritic and epibathyal settings can be assumed. *Costa* spp., an epi- to mesoneritic element, and *Paranesidea*? *brevis*, which seems to be absent in littoral and mixohaline habitats, are recorded in this assemblage more frequently. *Xestoleberis* spp. occurs commonly.

Thus, all assemblages range within the euphotic zone. While the FI, AX and AL assemblages are influenced by (storm) wave movements (epineritic zone; about < 40 m water depth; Liebau, 1980), the PC assemblage suggests a setting just below the storm wave base (upper infraneritic or mesoneritic zone, respectively; about > 40 m water depth; Liebau, 1980). All samples from drillings SW or directly upon the Mesozoic ridge (except sample HA 573/11) yielded epineritic ostracode assemblages, influenced by terrigenous influx (FI, AX) or not (AL). Slightly deeper, mesoneritic environments are only indicated by the PC assemblage found NE of the spur (HA 511) and in some samples of the more distally located well HA 66.

#### Salinity

The co-occurrence of coralline algal incrusted breccias/conglomerates at the transgression horizon and LBF clearly indicate full marine conditions already from the beginning of the transgression. The foraminiferal fauna and the corallinaceans occur throughout the drilled cores and point to a continuous fully marine environment. Also, the dinocysts *Nematosphaeropsis labyrinthus* and *Polysphaeridium zoharyi* indicate full marine conditions between c. 26 and 39 PSU (Soliman et al., 2022). This data is in accordance with the calculated salinity average of 35 PSU and a range between 31 and 40 PSU for the late Badenian of the Vienna Basin based on foraminifera assemblages (Kranner et al., 2021b). Only in HA 66 and HA 573 a distinct reduction in foraminifera, ostracode and dinoflagellate diversity points to a decrease in salinity at the Badenian/Sarmatian boundary.

The coccolith *Umbilicosphaera jafari* is relatively abundant in the studied three samples of HA 511. Although ecological information on this species is sparse it is nevertheless often interpreted as an indicator of elevated salinities (> 35 PSU) based on Wade and Bown (2006). Following this interpretation in the context of local conditions and paleotopography, the investigated samples in borehole HA 511 are reflective of variable surface water conditions, less influenced by both terrigenous influx and freshwater. Sample HA 511/11 is thus indicative of relatively high, potentially open marine salinities and likely warm temperatures.

Although fully marine conditions are proven throughout the upper Badenian succession, clear indications of freshwater input by fluvial action are documented by freshwater ostracodes and characeans (Danielopol et al., 1991; Gross, 2002).

The high percentage of allochthonous calcareous nannofossils and dinoflagellates in the samples SW of the Mesozoic ridge and the upper part of borehole HA 66 also indicates transport of reworked biota (see above). This freshwater input, however, did not markedly reduce overall salinity in the study area.

#### Substrate, water energy and turbidity

Overall, bottom conditions are highly variable in the studied sediments, what is already clearly expressed by the high number of facies present. These range from hard bottoms at the base of the upper Badenian successions with boulders, pebbles and gravels of the transgression horizons. These offer rocky or hard substrate for settlement by various sessile biota and even substrate for boring organisms like lithophagid bivalves and sponges. The corallinacean-dominated facies represent hard- to firmgrounds either with rhodolites or algal branches – “maërl”. These substrates are ideal for the settlement of cibicidid foraminifers (including *Lobatula lobatula*) but also for *Amphistegina* and small miliolids. In addition to abundant corallinacean grounds, also seagrass meadows may be present again indicated by abundant *Lobatula lobatula*. The latter occur isolated in fine grained sediment, and also ostracodes such as *Xestoleberis* (Gross, 2002) indicate a phytal habitat. The fine-grained siliciclastics (marl, calcareous marl, sandy marl) represent soft bottom substrates expressed by shallow infaunal foraminifers such as *Bolivina* and *Bulimina,* which indicate reduced oxygen conditions.

The water energy regime is also variable, with higher energy conditions at the transgression horizons and maybe stronger currents in the SW of the Mesozoic ridge due to fluvial input and coarser siliciclastics’ distribution. A major part of the successions can be considered to represent a relatively calm environment because of the high fine siliciclastic content. Also the limestones are mostly impure representing marly limestones or fine sandy limestones. Although rhodolites are frequently considered to form under higher energy conditions, this has been repeatedly falsified or some indications give even contradictory results (Aguirre et al., 2017). Only boxwork rhodolites with their thin corallinacean laminae and open spaced internal structure indicate quiet energy conditions. Since the majority of rhodolites belong into this category for major parts of the successions low energy conditions can be assumed. This low energy conditions are in contrast to the current-driven upper Badenian limestone close by in the NE part of the Leitha Mountains (Wiedl et al., 2014) what may be related to the platform character of the latter exposed to higher hydrodynamic action.

Overall, the high content of fine siliciclastics points at a high suspension load of the water column reducing light availability even in such a shallow water setting. The missing depth zonation both of LBFs and coralline red algae may be a reaction on this overall reduced transparency.

The moderately well-preserved nannofossil assemblages in the upper samples of HA 66 (HA 66/19, HA 66/8, HA 66/7) generally reflect relatively low coarse grained terrigenous influx and variable nutrient-rich conditions. These conditions are inferred by the common occurrence of small and medium-sized reticulofenestrids (*R. minuta*) with accessory *C. pelagicus*. Sample HA 66/19 contains common but only moderately well preserved 6-rayed discoasterids. These are commonly interpreted as indicators of more oligotrophic and warm surface water conditions with a deep nutricline (Bralower, 2002; Schueth & Bralower, 2015; Tangunan et al., 2018). Although this would point toward open marine conditions, isotopic data measured on discoasterids indicated that they may be able to also thrive in oligotrophic surface waters within the upper photic layer. Considering the low depth in which sediments of sample HA 66/19 were deposited, the occurrence of discoasterids likely indicates oligotrophic surface waters that intermittently supported a surface-dwelling stock of discoasterids in the late Badenian. In addition, the low coarser-grained terrigenous influx is also supported by low numbers of allochthonous taxa in this sample. In the upper samples HA 66/8 and HA 66/7, the content of terrigenous material increases, as indicated by higher numbers of allochthonous taxa. In addition, *Discoaster* spp. is replaced by *Helicosphaera* spp., which could reflect an increase of terrigenous influx and potentially lower salinity, as *Helicosphaera* spp., as well as small reticulofenestrids, can cope with variable salinity levels (Auer et al., 2014, 2015; Ćorić & Hohenegger, 2008; Lohmann & Carlson, 1981; Wade & Bown, 2006).

#### Temperature and climate

Best available temperature and climate proxies in the cores are the LBFs *Borelis*, *Amphistegina* and *Planostegina*. Today, *Borelis* is a circumtropical taxon having a lower temperature limit of 21.5 °C or 18 °C (Langer, 2008), *Heterostegina* (as proxy for *Planostegina)* occurs above 18 °C (Langer & Hottinger, 2000) and *Amphistegina* above the 14 °C winter isotherm (Langer, 2008). This allows a clear climatic assignment to tropical to warm-temperate conditions. Some dinoflagellate taxa such as *Melitasphaeridium choanophorum*, *Selenopemphix nephroides*, *Lingulodinium machaerophorum*, *Polysphaeridium zoharyi, Tuberculodinium vancampoae*, and *Tectatodinium pellitum* are also considered to be thermophilic (cf. Soliman et al., 2022).

All available data from the Vienna Basin point at a tropical to warm-temperate climate for the late Badenian. This is in contrast to the Badenian coralline algal limestones described by Randazzo et al. (1999) from the Pannonian Basin of Hungary. These are described as cool-water limestones, although nearly the same biota is recorded as in the Vienna Basin and therefore also clearly reflect a tropical to warm-temperate climate.

According to Kranner et al. (2021b), the calculated mean bottom water temperature (BWT) and sea surface temperature (SST) are both around 18 °C for the late Badenian in the Vienna Basin. The relatively high bottom and sea surface temperatures may be explained by a major shallowing trend from the middle to the late Badenian (Kranner et al., 2021a, b). These results are in contrast with data from Walbersdorf (Burgenland) in the Eisenstadt-Sopron Subbasin (of the Vienna Basin) where the rare occurrence of discoasterids was related to lower water temperature (Rögl & Müller, 1976) but north of Bratislava at Gajary discoasterids are well represented (Ozdínová, 2008). Diverging are also data from nearby of Bad Deutsch-Altenburg at Devínska Nová Ves reported by Kováčová et al. (2009), which point to a stratification of the water column and wider range in salinity. Similar indications are also reported from other parts of the Central Paratethys (e.g., Peryt et al., 2014). The reason for these differences may be due to the marginal marine settings of both study locations and, on the contrary, inclusion of a wide range of facies by Kranner et al., (2021a, b). The shallowing mentioned above may have overprinted the global cooling during the Middle Miocene Climatic Transition (MMCT) following the Middle Miocene Climate Optimum (MMCO) (Holbourn et al., 2005; Miller et al., 2020; Westerhold et al., 2020; Zachos et al., 2001). In the Pannonian Basin, however, oxygen stable isotope sediment contents indicate a distinct cooling gradient along the middle-upper Badenian transition (Báldi, 2006; Jiménez-Moreno et al., 2006; Mandic et al., 2019). This coincides well with the Mi-3 isotopic event and a global sea level rise of about 50 m (e.g., Miller et al., 2020). Comparing the Vienna Basin biota at large along this transition, a climatic decrease is very well represented. Contrary to the middle Badenian, no more coral reefs exist but only biostromal constructions (e.g., Wiedl, 2013), and also molluscs (Harzhauser & Piller, 2007) and the echinoid fauna (Kroh, 2007) show a clear reduction in species diversity. This decline is expressed in the mid-Badenian-extinction-event (MBEE) defined by Harzhauser and Piller (2007). Also pollen assemblages show a decrease in temperature but indicate still a subtropical climate (Doláková et al., 2020; Kováčová et al., 2011).

#### Paleogeography

The late Badenian transgression affected the Paratethys from the Vienna Basin to the Caspian Sea and also reflooded the Carpathian foredeep (Rögl, 1999). It is correlated to the 3^rd^ order sequence TB 2.5, which reflects a global sea level rise requiring an open seaway to (an)other marine basin(s).

The “Trans-Tethyan-Trench Corridor” (also: Slovenian Corridor, Trans-Tethyan Strait), the most important gateway between the Mediterranean and the Central Paratethys during the early to middle Badenian, was considered to be closed in the late Badenian (e.g., Harzhauser & Piller, 2007; Rögl, 1998; Studencka et al., 1998). Substantial water exchange was assumed between the Central and the Eastern Paratethys (e.g., Palcu et al., 2017; Popov et al., 2004), however, the biota of both realms is widely different and that of the Eastern Paratethys is strongly impoverished what impedes a major exchange (Harzhauser & Piller, 2007; Studencka et al., 1998). The basic questions therefor are: (i) was there a connection between the Central Paratethys and the Indo-Pacific, or/and (ii) did still a gateway exist between the Central Paratethys and the Mediterranean, and (iii) how strong was the exchange between the Central and the Eastern Paratethys? These basic questions have been reviewed and highlighted more recently in detail in respect to biota by Bartol et al. (2014) and regarding water balance by Palcu et al., (2015, 2017) and Simon et al. (2018).

(i) In the Romanian Transylvanian Basin and the Moravian-Polish-Ukrainian-Romanian Carpathian Foredeep radiolaria shales and pteropod marls occur in the upper Badenian (Dumitrică et al., 1975; Popescu, 1979). Pteropod marls are also reported from the Vienna Basin (Rögl & Müller, 1976) and even from core HA 20 of the Bad Deutsch-Altenburg-Hainburg area (studied herein for ostracodes) (Bohn-Havas & Zorn, 1994; Zorn, 1991). As a peculiarity, the pteropod fauna indicates a relationship to the North Sea Basin (Janssen & Zorn, 1993; Zorn, 1999). Rögl (1998) considered nannoplankton, diatoms and the radiolarian assemblages of the Central Paratethys to reflect a distinct Indo-Pacific character. Another Indo-Pacific relation seems to be documented by a fish fauna with the oldest parrot fish (*Calotomus preisli*) described by Bellwood and Schultz (1991) from the Vienna Basin. This oldest *Calotomus* species, however, may not indicate a migration from the Indo-Pacific into the Paratethys but represents an example of the opposite trajectory as shown in several other biota (Harzhauser et al., 2007).
  An open Indo-Pacific connection was reported from Georgia and the Transcaspian Basin during the late Badenian (=early Konkian) into the Eastern Paratethys, where marine conditions with stenohaline molluscs, echinoids, and foraminifera returned (Nevesskaya et al., 1986, 1987).
(ii) Studencka et al. (1998) reported that 26 bivalve species common to the Mediterranean Province, Atlantic Province and North Sea Basin appeared in the Central Paratethys together with the late Badenian transgression. These authors consider this appearance of newly evolved bivalve species (and other biota) a clear signal for a connection of the Central Paratethys with the Mediterranean. This view was reinforced by the data of Bartol et al. (2014), who studied, besides other biota, the gastropod species *Pereiraea gervaisi* in middle-late Badenian sections in Slovenia. This species does, however, occur not only in Slovenia but also in Croatia and Hungary, what points to a Mediterranean-Central Paratethys connection via the “Slovenian Corridor”. Also, the nannoplankton assemblage in Slovenia shows a strong similarity with the Mediterranean one, but that of the overall Central Paratethys indicates both Mediterranean and Eastern Paratethys relations.
(iii) The reduced salinity indicating middle Badenian (= Karaganian) biota of the Eastern Paratethys were influenced by Central Paratethyan marine biota at the base of the late Badenian (= lower Konkian). In the middle Konkian a great share of Eastern Paratethys biota occurs also in the Central Paratethys (Iljina, 2003; Studencka et al., 1998). The first information points to a west–east migration the second to an east–west trajectory. As connection between the Central and Eastern Paratethys during the late Badenian the Bârlad Strait north of Dobrogea through present-day Romania, Ukraine and Moldova is considered (Palcu et al., 2017; Popov et al., 2006; Simon et al., 2018; Studencka et al., 1998).

As a conclusion, the probably best-assured connection is that between the Central and the Eastern Paratethys via the Bârlad Strait. The water balance and the migration pathways changed even during this relatively short interval, and biotic exchange seems to have occurred in both directions (Palcu & Krijgsman, 2022; Palcu et al., 2015, 2017; Popov et al., 2006; Simon et al., 2018; Studencka et al., 1998). However, this connection does not explain the biotic assemblages in the Central Paratethys.

A direct connection between the Indo-Pacific and the Central Paratethys anticipated between the Black Sea plate and the Pontids (Rögl, 1998, 1999) is not substantiated by the described variety of biota. As Bartol et al. (2014, p. 153) pointed out, this interpretation is based merely on poor silicoflagellate and radiolarian assemblages which were studied only in a very limited part of Romania (Dumitrică et al., 1975) what cannot be considered representative for the entire Central Paratethys. Other biota cited as Indo-Pacific indicators are, e.g., larger benthic foraminifers (Rögl, 1998). Among them, the herein studied *Amphistegina* and *Planostegina* entered the Mediterranean already in the Early Miocene (e.g., Mandic & Piller, 2001; Rögl, 1998) and can therefor not considered Indo-Pacific migrants. Similar timing occurred in *Borelis* migrating into the Mediterranean during the Aquitanian (Bassi et al., 2021). In addition, Bassi et al. (2021, p. 397) state: “ … the westward migrants of *B. philippinensis* gave rise to *B. melo* (Aquitanian–Messinian) and *B. curdica* (Burdigalian–Tortonian). These two species became isolated from the Indo-Pacific by the eastern closure of the Mediterranean basin in Langhian times.” Fishes can also not be considered as cogent arguments for an Indo-Pacific influence (see above).

The mollusc data (Bartol et al., 2014; Studencka et al., 1998) clearly indicates that a Central Paratethys—Mediterranean seaway did exist with good faunal exchange during the late Badenian. The observations by Bartol et al. (2014) and also the distribution of larger benthic foraminifers (discussed above) and dinoflagellates (Soliman et al., 2022) strongly support that the Trans-Tethyan Corridor (Slovenian Corridor) was still active and allowed a biotic exchange and immigration of Mediterranean elements into the Central Paratethys. Another possible gateway between the late Badenian Paratethys Sea and the East Mediterranean was speculated through the Vardar/Axios Trench (North Macedonia to Thessaloniki) or Neo-Vardar Tectonic Zone (Hámor, 2002). The record is, however, vague and poorly constrained.

Overall, the available data reflects a complex pattern of biotic and hydraulic interrelationships and point to a mixture of Mediterranean and Eastern Paratethys elements during the late Badenian in the Central Paratethys.

## Conclusion

The study area at Bad Deutsch-Altenburg at the easternmost margin of the Vienna Basin shows a peculiar paleotopography which highly influenced and controlled the sedimentary depositional regime. The sediments of 10 investigated cores belong stratigraphically to the Serravallian (upper Middle Miocene). The major part represents upper Badenian (lower Serravallian) documented by foraminifera, calcareous nannoplankton, dinoflagellates and ostracodes, only in 2 drillings lower Sarmatian (upper Serravallian) is proven based on foraminifera and dinoflagellates. In terms of sequence stratigraphy the Badenian sediments represent the transgressive and highstand systems tract of 3^rd^ order sequence TB 2.5 (bound by the lowstands Ser 2 and Ser 3), the lower Sarmatian sediments can be correlated to sequence TB 2.6.

Paleotopography is marked by a Mesozoic ridge striking SE-NW and dipping basinwards. This ridge subdivided the sedimentary environment into a siliciclastic (sand) dominated in the SW and a carbonate dominated one in the NE. The coarse siliciclastics are fluvially delivered indicated by freshwater ostracodes and charophyte oogonias. The biotic fine fraction, represented by calcareous nannoplankton and dinoflagellates shows a high portion of allochthonous elements originating from Upper Cretaceous, Paleogene and Lower Miocene sources. The assocations in the SW of the ridge contain a very high share of allochthonous elements the carbonate sediments in the NE is mainly composed of autochthonous taxa.

The carbonates are highly variable representing 13 different facies mostly dominated by coralline algae in various growth forms but also by the larger benthic foraminifer *Amphistegina*. A clear distributional pattern of the carbonate facies was not detected neither areally nor temporally.

Sedimentation started with a transgressive conglomerate which shows already full marine conditions reflected by a foraminiferal fauna and boring biota such as marine bivalves (lithophagids) and sponges. Due to the dense drilling field and the recorded paleotopography water depth did not reach much more than 50 m. Water depth can also be estimated by LBF, coralline algae and ostracodes. Ostracodes allow a separation into 4 assemblages: the *Fabaeformiscandona-Ilyocypris* assemblage (FI), the *Aurila-Xestoleberis* assemblage (AX), the *Aurila-Loxocorniculum* assemblage (AL) and the *Pterygocythereis-Cytherella* assemblage (PC). Besides freshwater influx in FI assemblage all other assemblages reflect full marine conditions in an epi- to mesoneritic setting.

All studied biota (calcareous nannoplankton, coralline algae, foraminifers, ostracodes) indicates generally full marine salinity (of about 35 PSU) only in the area SW of the Mesozoic ridge riverine input may have reduced salinity. The Sarmatian sediments show reduced salinity conditions, in particular, indicated by the occurrence of a strongly reduced but specific foraminiferal fauna (e.g., *Saccammina sarmatica, Anomalinoides dividens*). Substrate conditions range from hardgrounds with gravelly bottom to coralline algal gravel and sand (phytal environment) to muddy softgrounds. All these offer divers ecological niches which are also reflected in the biotic assemblages. Due to the high pelitic content in most sediments turbidity can be considered generally high but, e.g., calcareous nannoplankton allows a more refined reconstruction. Water temperature reflects tropical to subtropical conditions clearly indicated by LBF (*Borelis, Amphistegina, Planostegina*) and rare corals. Although the sedimentary sequence was already deposited during the Middle Miocene Climate Transition (MMCT) the biota don’t reflect this cooling phase in the studied material. This can be explained by a shallowing of the depositional basin (Vienna Basin) and increasing restriction.

Paleogeographically, the recorded biota of the upper Badenian reflects a mixture of autochthonous Central Paratethyan elements but also a share of Mediterranean and Eastern Paratethyan origins. This can only be explained by an open connection to the Mediterranean via the Slovenian corridor on the one side and to the Eastern Paratethys via the Bârlad Strait on the other. A connection to the Indo-Pacific was only possible via the Eastern Paratethys and most so called Indo-Pacific biota migrated into the Central Paratethys from the Mediterranean Sea.

## Supplementary Information

**Additional file 1.** Studied drill sites and respective sample numbers (left column each), corresponding altitudes above sea level (asl), sampling depth and total depth of drilling below surface (bs) (right column each).

## Data Availability

All materials are deposited at the Institute of Earth Sciences, University of Graz; data and other details of this study are available from the corresponding author Werner E. Piller upon request (Additional file 1).
